# Developing a Dual-Targeting CD33/CD70 CAR T-Cell Strategy Against Acute Myeloid Leukemia to Overcome Tumor Heterogeneity

**DOI:** 10.3390/cancers18193183

**Published:** 2026-10-02

**Authors:** Emily C. Sullivan, Nabil Saleem, Areeba A. Hashmi, Kristopher A. Knight, Austre Y. Schiaffino Bustamante, Ahmed A. Aljudi, Brandon J. Fanelli, Sunil S. Raikar

**Affiliations:** 1 Department of Pediatrics, Emory University School of Medicine, Atlanta, GA 30322, USA; 2 Aflac Cancer and Blood Disorders Center, Children’s Healthcare of Atlanta, Atlanta, GA 30329, USA; 3 Molecular and Systems Pharmacology Graduate Program, Graduate Division of Biological and Biomedical Sciences, Emory University, Atlanta, GA 30322, USA; 4 Cancer Biology Graduate Program, Graduate Division of Biological and Biomedical Sciences, Laney Graduate School, Emory University, Atlanta, GA 30322, USA; 5 Department of Pathology, Children’s Healthcare of Atlanta, Atlanta, GA 30329, USA

**Keywords:** leukemia, immunotherapy, chimeric antigen receptor, CAR T cell, dual targeting, acute myeloid leukemia, CD33, CD70

## Abstract

Background: Acute myeloid leukemia (AML) remains the deadliest form of leukemia in children and adults despite therapeutic advances in recent decades. Chimeric antigen receptor (CAR) T-cell therapy is one such therapeutic approach that has been explored preclinically and clinically in AML; however, its efficacy is limited by antigen heterogeneity. To overcome this roadblock, we propose a dual-targeting CAR T-cell strategy, targeting the AML-associated antigens CD33 and CD70. Methods: To recapitulate tumor heterogeneity, we genetically edited the AML cell line Molm-13 using CRISPR-Cas9 to generate distinct sublines, namely CD33+/CD70−, CD33−/CD70+, and CD33+/CD70+. Two dual-targeting CAR T-cell approaches were developed: a “pooled” product, in which CAR T cells directed towards CD33 or CD70 are combined and co-administered, and a “co-transduced” product, wherein one population of T cells is transduced with both CD33-CAR- and CD70-CAR-encoding viral vectors. Results: Pooled and co-transduced products demonstrated potent cytotoxicity against all Molm-13 populations, whereas single-antigen CAR approaches were not as effective when their respective target antigen was absent. Mice engrafted with CD33/CD70 heterogeneous Molm-13 cells were treated with pooled or co-transduced CD33/CD70 CAR T cells. Both groups exhibited a significant survival advantage over the untreated group, with the co-transduced approach showing a statistically significant advantage over the pooled group. Conclusions: In summary, engineering one T cell to express both CARs yielded superior cytotoxicity when targeting a heterogeneous AML blast population.

## Simple Summary

Relapsed/refractory acute myeloid leukemia (AML) remains difficult to treat in both pediatric and adult patients. Chimeric antigen receptor (CAR) T-cell therapy provides a promising treatment strategy for treating this patient population; however, antigen heterogeneity remains a key obstacle in CAR T-cell success for AML. We generated dual-targeting pooled and co-transduced CAR T-cell products against the AML-associated antigens CD33 and CD70 and evaluated their cytotoxicity against a CD33/CD70 heterogeneous model of AML.

## 1. Introduction

Acute myeloid leukemia (AML) is the most prevalent acute leukemia in adults and the second-most prevalent acute leukemia in children after B-cell acute lymphoblastic leukemia (B-ALL) [1,2]. AML is regarded as the deadliest leukemia in both adults and children, with 5-year overall survival rates ranging from 30–50% in adults to 50–70% in children [3,4,5]. Poor outcomes can be due to treatment failure from induction chemotherapy as well as high rates of relapse [3,6,7,8]. The current standard of care for relapsed/refractory (R/R) AML is an allogeneic hematopoietic stem cell transplant (allo-HSCT), which offers the potential for cure. However, a limitation of allo-HSCT is the need for complete remission prior to transplant, and nearly half of R/R AML patients fail to achieve this after reinduction chemotherapy [9,10,11]. This highlights an urgent clinical need for new therapeutic strategies capable of bridging patients to transplant and ultimately improving survival outcomes in AML.

Chimeric antigen receptor (CAR) T-cell therapy involves genetically modifying human T cells to express a receptor that binds to an antigen found on the surface of cancer cells and initiates T-cell-mediated cytotoxicity [12,13]. This state-of-the-art immunotherapy was first to be FDA approved for R/R B-ALL in 2017, and in recent years, it has been approved for other cancers, including large B-cell lymphoma, small lymphocytic leukemia, chronic lymphocytic leukemia, and multiple myeloma [14,15,16,17,18,19,20]. As a result, CAR T-cell therapy has served as an invaluable tool in inducing remission for patients with R/R malignancies that have homogenous surface markers.

In AML, several groups have shown limited preclinical and clinical success using CAR T cells against CD123, CLL-1, CD33, and CD70; however, this success pales in comparison to that of CD19-CAR T cells in B-cell malignancies [21,22,23,24,25,26,27,28,29,30,31]. This failure can be attributed to the antigen heterogeneity seen in myeloid-lineage diseases [32]. In contrast with B-ALL, AML blasts do not all share one homogenous surface marker, limiting the success of antigen-targeting therapies, such as CAR T cells [21,33,34]. Recently, dual-CAR T cells have emerged as a promising tool to overcome antigen heterogeneity and antigen escape in AML [35]. Most dual-targeting CARs fit into the confines of Boolean logic, which represents algebraic processes wherein outcomes can be classified as “OR”, “AND”, or “NOT”. In the scenario of dual CARs, each outcome represents the potential for T-cell activation upon binding, or lack thereof, to antigen. The most commonly utilized dual-CAR strategy in clinical trials remains an “OR” approach, wherein two different CARs are expressed on different or the same T cell, and activation of either CAR alone triggers cancer cell death. Prior and existing clinical trials in leukemia utilize a “pooled” or “co-administered” approach (NCT04340154, NCT05674175, NCT04204161) in which two separate CAR T cells are administered simultaneously or sequentially, while others aim to highlight the advantages of a “co-transduced” approach (NCT02443831, NCT03330691, NCT03919526), wherein one CAR T cell expresses both CARs [21,35]. Regardless of structure, multiple antigen combinations have been identified for AML through transcriptomic and flow cytometric analyses. CD33 and CD70 form one of these combinations that has yet to be evaluated extensively in a dual-CAR T-cell approach against AML [36].

CD33 is a sialic acid binding immunoglobulin-like lectin with inhibitory functions, while CD70 is a tumor necrosis factor superfamily ligand that promotes lymphocyte activation [29,30,37]. Perna et al. have demonstrated that the surface expression of CD33 or CD70 is significantly higher than the surface expression of either antigen alone on primary AML patient samples, making these targets ideal for a multivalent CAR T-cell approach [36,37]. A dual-targeted approach using CD70-CAR T cells that secrete a CD33/CD3-directed bispecific T-cell-engaging antibody molecule has demonstrated increased killing of AML blasts when compared to single-target approaches; this is encouraging, as it relates to the dual targeting of CD33 and CD70 [38]. However, the use of BiTEs has been shown to produce T-cell exhaustion in a number of diseases [39,40,41]. Furthermore, the use of bispecific T-cell engagers and CAR T cells targeting the same antigen has been evaluated directly by Rohrbacher et al. in AML, with this group noting that FLT3-directed BiTEs were inferior to CAR T cells directed towards the same antigen [41]. Herein, we present a novel AML treatment approach comprising a dual-targeting CAR T cell directed against CD33 and CD70. In this preclinical study, we evaluated dual-targeting CAR T-cell strategies compared to their single-targeting counterparts *in vitro*, and we compared two commonly used dual-targeting “OR” CAR products head-to-head *in vivo* to elucidate the optimal dual-targeting CD33/CD70-CAR product against AML.

## 2. Methods

### 2.1. CD33/CD70 Gene Expression in AML

The publicly available BloodSpot and St. Jude PeCan databases were mined for RNA expressions of CD33 and CD70, stratified by diagnosis vs relapse and by genetic abnormalities. Both adult and pediatric AML bone marrow samples were used in the analysis. Data are reported as Log_2_ FPKM (fragments per kilobase of transcript per million mapped fragments) or Log_10_ MoR (Mean of Ratios) [42,43].

### 2.2. Cell Lines

Molm-13, MV4-11, and Nomo-1 cell lines purchased from American Type Culture Collection (ATCC, Manassas, VA, USA) were maintained in RPMI (Corning, Manassas, VA, USA) supplemented with 10% fetal bovine serum (FBS, Corning, Woodland, CA, USA) and 1% penicillin–streptomycin (Life Technologies, Waltham, MA, USA) according to ATCC guidelines. Cultures were incubated at 37 °C with 5% CO_2_.

### 2.3. Primary AML Samples

A total of three different AML patient samples were used in this study. These viably frozen de-identified pediatric AML samples were obtained from the Leukemia/Lymphoma Biorepository at the Aflac Cancer and Blood Disorders Center at Children’s Healthcare of Atlanta (CHOA). Patients and/or their legal guardians provided written informed consent that permitted the use of biological material in accordance with a protocol that was approved by CHOA’s Institutional Review Board (IRB) (Protocol #2025P012283). The specific AML samples were chosen as the blast percentage of each sample was >90%. We excluded patient samples that had a diagnosis of acute promyelocytic leukemia (APL; French–American–British classification M3) [44]. All samples were thawed simultaneously, and post-thaw blast viability was maintained above 85% for all samples used. Cytogenetic and molecular features of these cases are shown in Table S1.

### 2.4. Flow Cytometry

All flow cytometry was performed using the Cytek Aurora spectral flow cytometer (Cytek Biosciences, Fremont, CA, USA). Samples were stained with the respective fluorophore-conjugated antibodies, resuspended in FACS buffer (1× PBS with 2.5% FBS), and passed through the flow cytometer. Forward and side scatter voltages were set between 50 and 200, and autofluorescence was automatically subtracted during the spectral unmixing process. Data were analyzed using FlowJo (Version 10.10.0, BD Biosciences, Franklin Lake, NJ, USA).

### 2.5. CD33/CD70 Surface Expression

Primary AML samples obtained from our institutional biorepository and AML cell lines were washed once with FACS buffer and stained with eFluor780 viability dye (Thermo Fisher Scientific, Waltham, MA, USA), anti-CD33-FITC (BioLegend, San Diego, CA, USA), and anti-CD70-Pe-Cy7 (BioLegend, San Diego, CA, USA) and then diluted 1:50 in FACS buffer and incubated on ice for 30 min. Primary AML cells were additionally stained with CD45-BV650 (BioLegend, San Diego, CA, USA). Cells were subsequently washed once more with FACS buffer and underwent flow cytometry via the Cytek Aurora. All results were analyzed using FlowJo (Version 10.10.0).

### 2.6. In Vitro Cytotoxicity Assays

WT, CD33 KO, and CD70 KO Molm-13 cells (target: T) were stained with VPD450 intracellular dye (BD, Franklin Lakes, NJ, USA) according to the manufacturer’s protocol and incubated with CAR T cells (effector: E) at various E:T ratios. Cells were co-cultured for 24 h at 37 °C with 5% CO_2_. Co-cultures were subsequently washed with Annexin V Binding Buffer (0.025 mM calcium chloride + 1.4 mM sodium chloride + 0.1 mM HEPES) and stained with eFluor780 viability dye (Thermo Fisher Scientific, Waltham, MA) and annexin V-APC (BioLegend, San Diego, CA, USA) before being diluted 1:50 in Annexin V Binding Buffer for 30 min on ice. Cells were once more washed with Annexin V Binding Buffer and underwent flow cytometry using the Cytek Aurora. Stopping gates were set at 20 μL per sample for all samples so that the raw live cell numbers could be calculated (volumetric collection) [45].

### 2.7. CAR T-Cell Immunophenotyping

To assess potential differences in the phenotypic landscape of our individually transduced pooled CAR T-cell product vs our co-transduced CAR T-cell product, we isolated T cells from healthy human donors and expanded them ex vivo for 7 days. On day 7, CAR T cells were washed with FACS buffer and stained with eFluor780 viability dye (Thermo Fisher Scientific, Waltham, MA, USA) and an antibody cocktail (diluted 1:50 in FACS buffer) consisting of T-cell-lineage surface markers, including CD3-FITC (BD, Franklin Lakes, NJ, USA), CD4-Pacific Blue (BioLegend, San Diego, CA, USA), CD8-PerCP (BioLegend, San Diego, CA, USA), and (G4S)_3_ Linker-PE (Cell Signaling, Danvers, MA, USA), to determine CAR expression. They were additionally stained with the following T-cell phenotype markers: CD45RA-Alexa Fluor 647, CCR7-BV650, and PD-1-PE-Cy7 (all from BioLegend, San Diego, CA, USA) [46]. Cells were incubated on ice for 30 min in the dark. Cells were washed once more with FACS buffer and underwent flow cytometry.

### 2.8. Intracellular Flow Cytometry

Molm-13 target cells were stained with VPD450 (BD, Franklin Lakes, NJ, USA) according to the manufacturer’s protocol and co-cultured at a 1:1 ratio with the respective CAR T cells for 20 h at 37 °C with 5% CO_2_, followed by the addition of BD GolgiStop (BD, Franklin Lakes, NJ, USA) and a further 4 h of incubation. Cells were washed with FACS buffer and stained with eFluor780 viability dye (Thermo Fisher Scientific, Waltham, MA, USA) and CD69-BV605 (BioLegend, San Diego, CA, USA) for 30 min on ice. Cells were washed twice with FACS buffer prior to fixation and permeabilization using BD Fixation/Permeabilization solution according to the manufacturer’s protocol, followed by incubation at 4 °C for 20 min. Cells were then washed twice with BD Perm/Wash buffer (BD, Franklin Lakes, NJ, USA). Permeabilized cells were stained with an antibody cocktail comprising interleukin-2-FITC (Invitrogen, Waltham, MA, USA), granzyme B-APC (BioLegend, San Diego, CA, USA), and tumor necrosis factor-α-PE (BioLegend, San Diego, CA, USA) in BD Perm/Wash buffer and incubated on ice for 30 min. Subsequently, cells were washed twice with BD Perm/Wash buffer and resuspended in FACS buffer prior to acquisition.

### 2.9. CRISPR-Cas9 Editing

Cas9 enzymes were purchased from Integrated DNA Technologies (IDT, Coralville, IA, USA). Single-guide RNAs (sgRNAs) against CD33 and CD70, as described by Silva et al., were purchased from IDT [38]. The sequences from 5′ to 3′ were as follows: CD33 sgRNA—GTCAGTGACGGTACAGGA, and CD70 sgRNA—GAGCTGCAGCTGAATCACAC. Briefly, 5 × 10^6^ Molm-13 AML cells were resuspended in 5 mL of opti-MEM reduced serum media (Gibco, Waltham, MA, USA) and transferred to a 0.2 cm electroporation cuvette. In total, 10 μg of Cas9 enzyme and 5 μg of each respective sgRNA were allowed to complex at room temperature for 10 min prior to being added to cuvettes and electroporation was achieved using square wave settings of 250 V, 5 ms, 1 pulse in a Bio-Rad Gene Pulser X cell system (Bio-Rad, Hercules, CA, USA). Electroporated cells then underwent fluorescence-activated cell sorting via Emory + Pediatrics/Winship ARIA II to isolate the CD33 knockout (CD33 KO) or CD70 knockout (CD70 KO) Molm-13 sublines.

### 2.10. CAR Design

The binding domain of our CD33-CAR is derived from the CD33-directed M195 antibody’s heavy and light chain sequences, while the binding domain of our CD70-CAR is derived from WO2014158821A1 [47,48]. All sequences were codon optimized for optimal human expression using IDT’s codon optimization tool. Variable heavy (V_H_) and light (V_L_) chains for each binding domain were connected by a (G4S)_3_ linker and cloned into a plasmid backbone containing an EF1α promoter—generously donated from H. Trent Spencer, PhD, which already contained sequences corresponding to human CD8α/CD28 hinge, CD28 co-stimulatory, and CD3ζ activation domains—to create a cohesive second-generation CAR sequence. High-titer lentiviral vector for each construct was subsequently generated by VectorBuilder (VectorBuilder, Chicago, IL, USA).

### 2.11. Primary T-Cell Cultures and Generation of CAR T Cells

Peripheral blood from healthy adult volunteers was obtained and peripheral blood mononuclear cells (PBMCs) were isolated from donor blood via Ficoll-Paque (Cytiva, Marlborough, MA, USA) density gradient centrifugation. T cells were isolated from the resulting PBMC population using a magnetic T cell Isolation kit (StemCell, Vancouver, BC, Canada); resuspended in X-VIVO 15 media (Lonza, Walkersville, MD, USA); supplemented with 10% fetal bovine serum (FBS, Corning, Woodland, CA, USA), 1% penicillin–streptomycin (Life Technologies, Waltham, MA, USA), 100 IU/mL interleukin-2 (Peprotech, Cranbury, NJ, USA), and 100 IU/mL interleukin-7 (Peprotech, Cranbury, NJ, USA); and stimulated with CD3/CD28 Dynabeads (Thermo Fisher Scientific, Waltham, MA, USA) for 48 h. Subsequently, the beads were removed, and the T cells were transduced by lentiviral vectors encoding our CARs at a predetermined multiplicity of infection (MOI) with polybrene (VectorBuilder, Chicago, IL, USA). The lentiviral vector was allowed to incubate with T cells for 18 h, after which the vector was removed; the T cells were then placed at a concentration of 1 × 10^5^ cells/mL in supplemented X-VIVO 15 media and allowed to expand for 5 days. All incubations were performed at 37 °C with 5% CO_2_.

### 2.12. Pooled and Co-Transduced CAR T-Cell Generation

Our pooled CAR T-cell product comprised CAR T cells independently transduced with CD33-CAR- *or* CD70-CAR-encoding lentiviral vectors and combined in a 1:1 ratio. Our co-transduced (Co-T) CAR T-cell product was developed by transducing T cells simultaneously with CD33-CAR and CD70-CAR lentiviral vectors.

### 2.13. In Vivo Studies

Male and female NOD. *Cg-Prkdc^scid^* Il2rg^tw1Wjl^/SzJ (NSG) mice were purchased from Jackson Laboratories (Bar Harbor, ME, USA) and housed in cages of up to 5 mice per cage in a sterile environment. A total of approximately 40 mice were used throughout the course of this manuscript, with no less than 6 mice per experimental group. Sample sizes were determined using the GraphPad Sample Size and Power Analysis software (GraphPad Software Inc., Boston, MA, USA). No a priori exclusion criteria were set beyond an age limit of 8–12 weeks. All mice were housed in the same room, provided the same food and water, and received cage changes on the same days. For all studies, an additional lab member not directly conducting the studies was aware of group allocation while main researchers conducting the experiments were not until mice reached endpoint naturally or a humane endpoint. Evaluations were made on a scale of 1–4 (a score of 1 indicating “normal,” a score of 2 indicating “slightly abnormal,” a score of 3 indicating “moderately abnormal,” and a score of 4 indicating “severely abnormal”) for coat appearance, posture, motility, activity level, and body weight. A total score of 4 or a weight loss of 20% or greater triggered immediate euthanasia to reach a humane endpoint. Additionally, any pre-morbid mice were sacrificed at the recommendation of the veterinary staff.

Single-targeting CAR *in vivo:* NSG mice, aged between 8 and 12 weeks, were intravenously injected on day −4 with 5 × 10^5^ luciferase-expressing Molm-13 cells, of which 1/3 were CD33 KO, 1/3 were CD70 KO, and 1/3 were WT. On day 0, mice were intravenously injected (*n* = 6 per group) at random with either PBS, 1 × 10^6^ CD33-CAR T cells or 1 × 10^6^ CD70-CAR T cells. Upon endpoint (significant weight loss, pallor, and hind limb paralysis), mice from each group were dissected, following which the spleen and bone marrow were harvested and stained with eFluor780 viability dye (Thermo Fisher Scientific, Waltham, MA, USA), anti-CD45-BV650 (BioLegend, San Diego, CA, USA), anti-CD3-FITC (BD, Franklin Lakes, NJ, USA), anti-(G4S)_3_-PE (Cell Signaling, Danvers, MA, USA), anti-CD33-APC (BD, Franklin Lakes, NJ, USA), and anti-CD70-Pe-Cy7 antibodies (BioLegend, San Diego, CA) before being analyzed via flow cytometry.

Dual-targeting CAR *in vivo:* NSG mice, aged between 8 and 12 weeks, were intravenously injected on day −4 with 5 × 10^5^ CD33/CD70 heterogeneous luciferase-expressing Molm-13 cells. On day −1, mice were intraperitoneally injected with 3 mg (200 μL of a 15 mg/mL PBS solution) of D-luciferin (Revvity, Waltham, MA, USA) and imaged using an In Vivo Imaging System (IVIS, Perkin Elmer, Waltham, MA, USA) to confirm leukemia engraftment. On day 0, mice were intravenously injected with either PBS, 1 × 10^6^ pooled CAR T cells, or 1 × 10^6^ co-transduced CAR T cells (*n* = 6 per group) at random. On days 6 and 13, mice were once more intraperitoneally injected with D-luciferin and imaged via IVIS. Bioluminescence was analyzed via Living Image Software (Revvity, Waltham, MA, USA) and survival was tracked.

### 2.14. Statistical Analysis

All experiments have sound controls and adequately powered groups calculated via the GraphPad Sample Size and Power Analysis software (GraphPad Software Inc., Boston, MA, USA). Data are presented as mean ± standard deviation. A one-way or two-way ANOVA followed by a post hoc Tukey’s test was used to calculate *p*-values for experiments with more than two groups when appropriate, depending on the number of independent variables. Kaplan–Meier curves were used for survival analysis where appropriate. Statistical analysis of survival curves was conducted using a Log-Rank (Mantel–Cox) test with Bonferroni correction. For all *in vivo* studies, both male and female mice were used to control for sex as a biological variable. Appropriate statistical tests were performed by Prism 10 (GraphPad Software Inc, Boston, MA), and statistical significance was achieved at *p* < 0.05.

## 3. Results

### 3.1. CD33 and CD70 Transcriptomic and Surface Expression in AML

**Figure 1:**
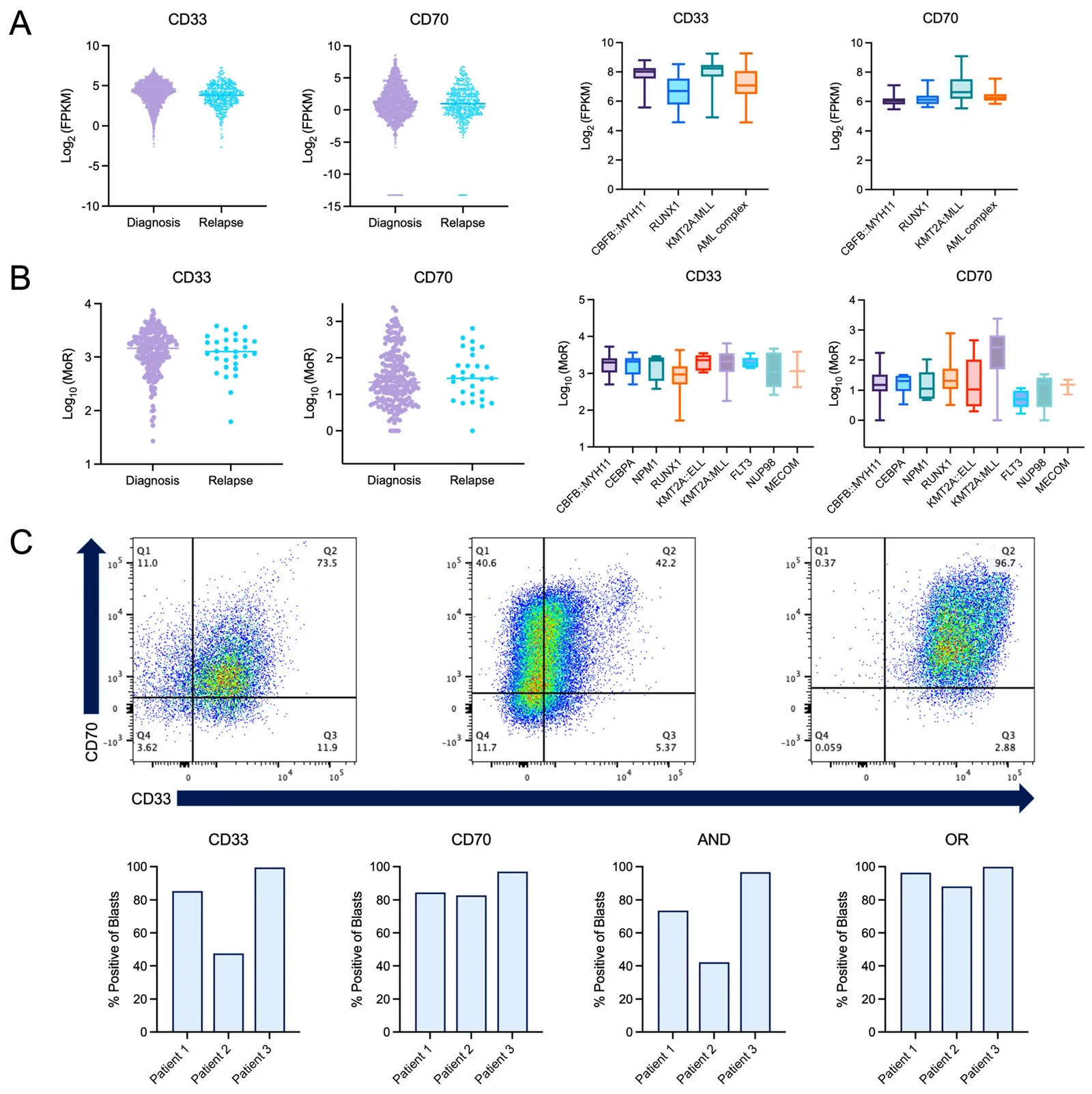
CD33 and CD70 expression in adult and pediatric AML. (**A**) Gene expression analysis using RNASeq data from *BloodSpot* shows positive but variable expression of CD33 and CD70 in over 1500 AML patient samples at both diagnosis and relapse, regardless of genetic abnormality. Error bars represent SD. (**B**) CD33 and CD70 expression in pediatric AML samples derived from bulk RNASeq data from the *St. Jude PeCan* database, showing positive but varied expression of both genes at diagnosis and relapse. Heterogeneous expression is seen across different AML subtypes classified by genetic abnormality. (**C**) Plots depicting CD33 and CD70 surface expression via flow cytometry of AML patient samples obtained from our institutional biorepository.

To confirm CD33 and CD70 expression on AML cells and establish both antigens as viable CAR targets, we analyzed healthy and malignant patient samples using various techniques. Bulk RNA sequencing of over 1500 adult AML samples from the publicly available BloodSpot database (Figure 1A), as well as over 200 pediatric AML samples from the *St. Jude PeCan* database (Figure 1B), showed positive, albeit varied, gene expression of CD33 and CD70 at both diagnosis and relapse, regardless of genetic abnormality. To characterize the surface expression of CD33 and CD70, we analyzed three de-identified AML patient samples obtained through our institutional biorepository (Table S1) and found heterogeneous expression of CD33 and CD70 surface markers on all samples. Importantly, the expression of CD33 *or* CD70 exceeded that of either antigen alone, suggesting that an “OR” CAR strategy is optimal to treat AML (Figure 1C and Figure S1). These findings support our hypothesis that a single-targeting CAR approach directed towards either CD33 or CD70 is less likely to effectively kill AML blasts *in vitro*, and a dual-targeting CAR strategy, targeting both CD33 and CD70, is likely to be more cytotoxic against AML blasts *in vitro*.

### 3.2. In Vitro AML Cell Line Models Are Not Entirely Representative of Primary Patient Blasts

**Figure 2:**
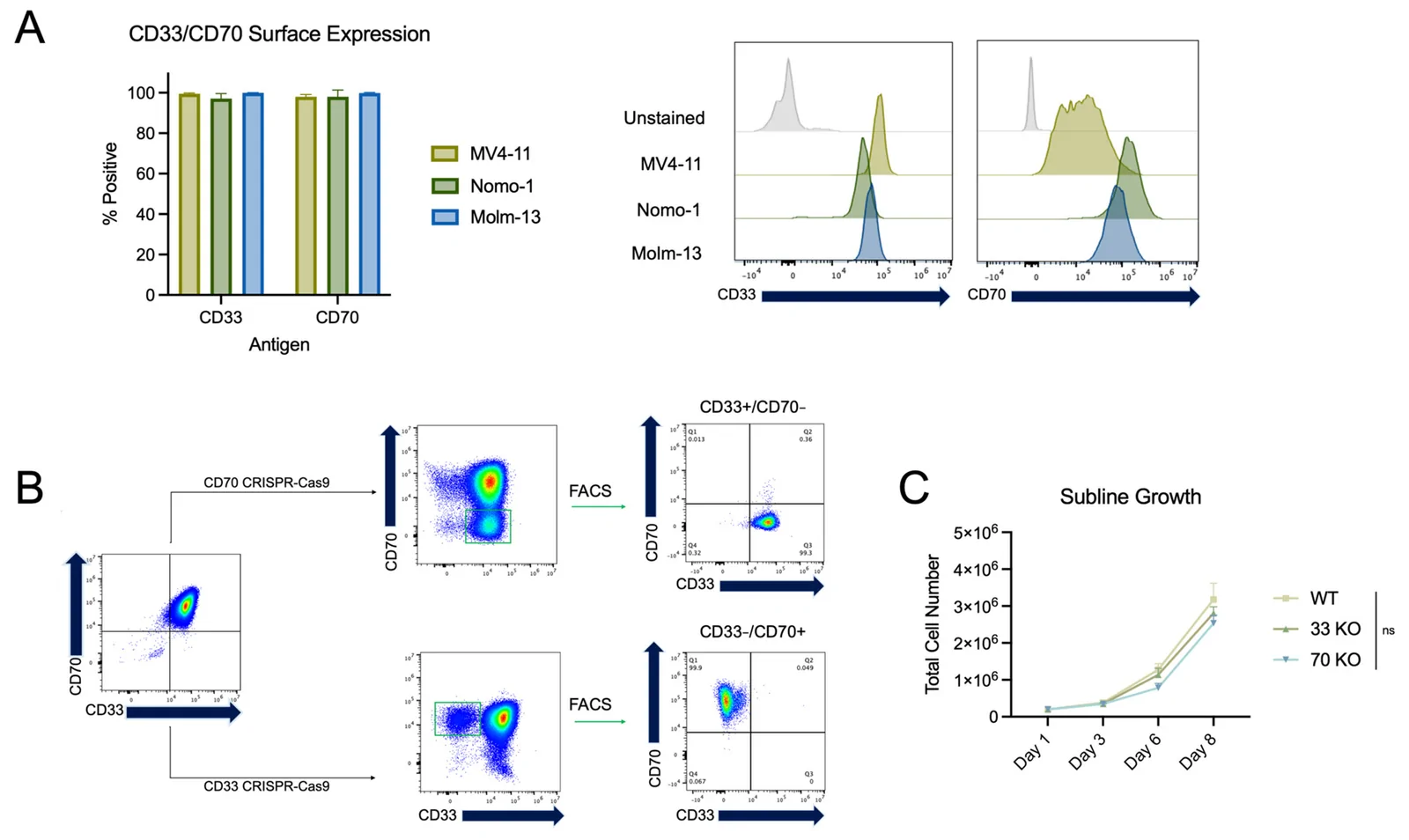
Generation of a CD33/CD70 heterogeneous AML cell line. (**A**) CD33 and CD70 surface expression of the widely established AML cell lines MV4-11, Nomo-1, and Molm-13, followed by representative histograms of each. Error bars represent SD, *n* = 3 experimental replicates. Statistical analysis for differential expression was done using a two-way ANOVA with a post hoc Tukey’s test and was non-significant (ns). (**B**) Flow plots showing Molm-13 cells before and after undergoing CRISPR-Cas9 editing to knockout (KO) CD33 or CD70 expression, followed by fluorescence-activated cell sorting (FACS). (**C**) Trypan blue exclusion assay determining the growth of wildtype (WT), CD33 KO (33KO), or CD70 KO (70KO) Molm-13 sublines post-CRISPR. Error bars represent SD, *n* = 3 experimental replicates. Statistical analysis represents one-way ANOVA with a post hoc Tukey’s test (ns—non-significant).

Commonly used AML cell lines show near 100% positivity for both CD33 and CD70 surface markers via flow cytometry, suggesting that they do not accurately recapitulate the tumor heterogeneity seen in patient samples (Figure 2A and Figure S2). To address this deficit, we generated CD33- or CD70-negative Molm-13 cells via CRISPR-Cas9 technology. We chose the AML cell line Molm-13 due to its established success in murine xenograft models [49]. Using Cas9 endonuclease and single-guide RNAs (sgRNAs) directed towards the CD33 or CD70 genes, we knocked out either CD33 or CD70 in wildtype (WT) Molm-13 cells and subsequently utilized fluorescence-activated cell sorting to isolate three unique Molm-13 sublines: CD33−/CD70+ (CD33 KO), CD33+/CD70− (CD70 KO), and CD33+/CD70+ (WT) (Figure 2B). To evaluate whether one subline outcompeted the others, we measured growth of each subline via trypan blue exclusion assay for one week and did not see significant differential growth rates (Figure 2C). In an alternative approach, WT Molm-13 cells were flow sorted into CD33/CD70-low populations based on median fluorescence intensity (MFI); however, expression of both antigens did not remain constant over time (Figure S3). Therefore, we chose to move forward with our KO approach.

### 3.3. Targeting CD33 and CD70 Concurrently Effectively Kills Heterogeneous AML Blast Populations

**Figure 3:**
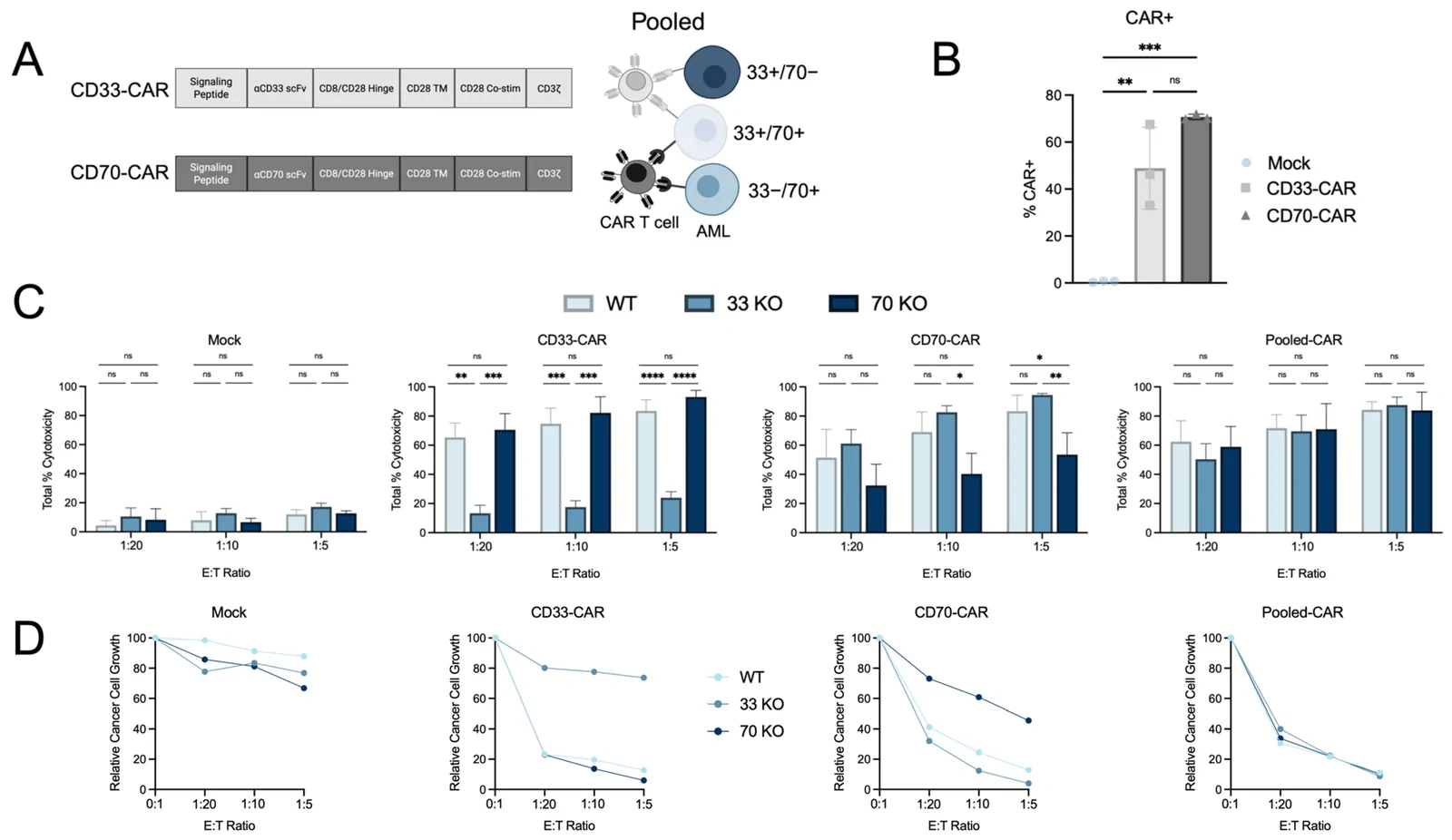
Single and pooled CAR T-cell product designs and cytotoxic efficacy *in vitro*. (**A**) Schematic of individual CD33-CAR and CD70-CAR constructs, including scFv antigen binding domains and hinge, transmembrane, co-stimulatory, and intracellular signaling domains. (**B**) CAR-positive cell percentage following lentiviral transduction, as measured by flow cytometry using an antibody against the (G4S)_3_ linker contained in both scFvs. Error bars represent SD, *n* = 3 experimental replicates, three biological donors. Statistical analysis represents one-way ANOVA (*** *p* < 0.001; ** *p* < 0.01). (**C**) Flow cytometry-based 24 h cytotoxicity assays of single- and dual-targeting CAR T cells after co-incubation with WT, CD33 KO, or CD70 KO Molm-13 AML target cells at a range of effector-to-target (E:T) ratios. Error bars represent SD, *n* = 3 experimental replicates, three biological donors. Statistical analysis represents one-way ANOVA with a post hoc Tukey’s test (**** *p* < 0.0001; *** *p* < 0.001; ** *p* < 0.01; * *p* < 0.05). (**D**) Mean plots of relative cell growth of WT, CD33 KO, or CD70 KO Molm-13 AML target cells at a range of E:T ratios. *n* = 3 experimental replicates, three biological donors. Individual donor plots are shown in Figure S4.

CAR sequences comprised either a CD33-targeting or CD70-targeting single-chain variable fragment (scFv) attached to a CD8α-CD28 hybrid hinge, CD28 co-stimulatory, and CD3ζ domain (Figure 3A). Primary human T cells were isolated from peripheral blood mononuclear cells (PBMCs) and stably transduced with lentiviral vector corresponding to each CAR. CAR expression, measured via flow cytometry using a monoclonal antibody specific to the linker sequence of each scFv, was comparable for CD33-CAR and CD70-CAR T cells (Figure 3B and Figure S4). Single-targeting CD33-CAR and CD70-CAR T cells were combined in a 1:1 ratio to comprise our “pooled” CAR T-cell product. To assess the cytotoxicity of each CAR product against all of our AML sublines (WT, CD33 KO, and CD70 KO), single and pooled CAR T cells (effector: E) and AML cells (target: T) were co-cultured for 24 h at various effector-to-target (E:T) ratios and analyzed via flow cytometry for expression of the apoptotic marker annexin V and the dead cell marker efluor780. Cytotoxicity was assessed through two methods of analysis: (1) percentage of dead target cells relative to the untreated group, and (2) enumeration of live target cells relative to the untreated control group. All CAR strategies were compared to untransduced T-cell controls (“mock”), which exhibited negligible killing of AML blasts. When the target antigen of a particular CAR T-cell product was absent from the cell surfaces of the AML blast population, the corresponding CAR T-cell product had significantly lower cytotoxicity *in vitro*. For example, our CD33-CAR was able to effectively kill WT and CD70 KO blasts but was ineffective against CD33 KO blasts. Similarly, our CD70-CAR was able to effectively kill WT and CD33 KO blasts but was not as cytotoxic against CD70 KO blasts. Importantly, our pooled CAR approach was able to effectively kill all three blast populations, suggesting that targeting both antigens is necessary to effectively treat AML with heterogeneous antigen expression (Figure 3C and Figure S5). When analyzing target cell growth, our CD33-CAR was only able to significantly inhibit the growth of WT and CD70 KO blasts, while our CD70-CAR was only capable of inhibiting the growth of WT and CD33 KO blasts; however, our pooled CAR was able to inhibit growth of CD33+/CD70−, CD33−/CD70+, and CD33+/CD70+ blasts (Figure 3D and Figure S6).

### 3.4. Pooled and Co-Transduced Dual-Targeting CD33/CD70 CAR T-Cell Strategies Exhibit Comparable In Vitro Cytotoxicity

**Figure 4:**
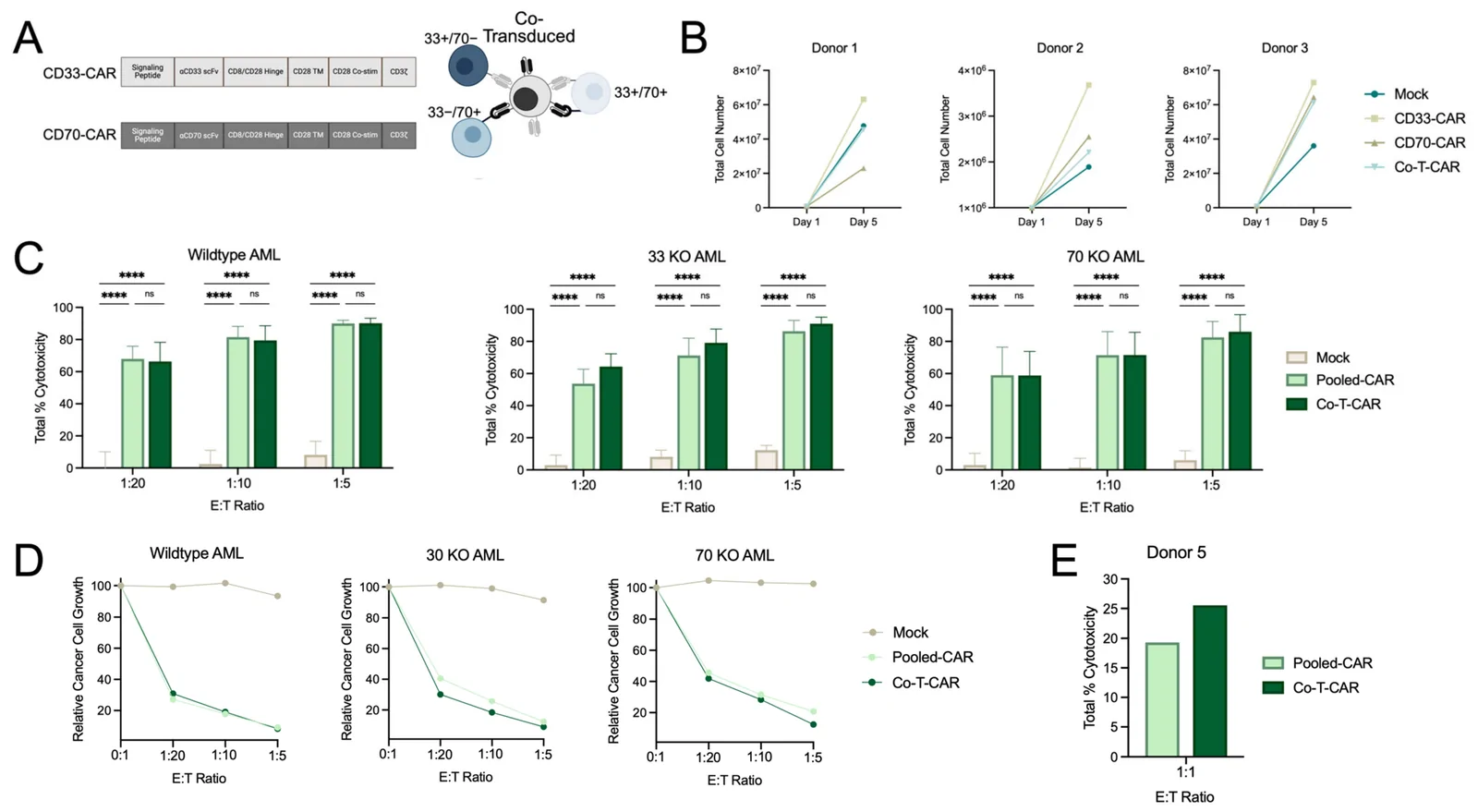
Co-transduced CAR T-cell product design and cytotoxic efficacy *in vitro*. (**A**) Schematic of individual CD33-CAR and CD70-CAR constructs that were used to simultaneously transduce a single population of primary T cells to create a co-transduced CAR T-cell product (Co-T-CAR). (**B**) CAR T-cell growth over five days post-transduction during the manufacturing process. *n* = 3 experimental replicates, three biological donors. (**C**) Flow cytometry-based 24 h cytotoxicity assays of pooled and co-transduced CAR T cells after co-incubation with WT, CD33 KO, or CD70 KO Molm-13 AML target cells at a range of E:T ratios. Error bars represent SD, *n* = 3 experimental replicates, three biological donors. Statistical analysis represents a one-way ANOVA with a post hoc Tukey’s test (**** *p* < 0.0001; ns). (**D**) Representative plots of relative cell growth of WT, CD33 KO, or CD70 KO Molm-13 AML target cells at a range of E:T ratios. *n* = 3 experimental replicates, three biological donors. (**E**) A primary AML patient sample obtained from our institutional biorepository was co-incubated with dual-CAR T-cell products separately in a 4 h flow cytometry-based cytotoxicity assay. AML blasts were gated in by assessing CD45 dim and side scatter patterns, and cytotoxicity was measured across both experimental groups. Mock T-cell cytotoxicity was subtracted from the reported cytotoxicity of pooled CAR and Co-T-CAR to normalize each group to account for mock T-cell cytotoxicity.

Although our pooled CAR strategy proved cytotoxic against heterogeneous AML, we sought to compare it to a streamlined co-transduced approach in which both CD33-CARs and CD70-CARs were expressed on the same T cell (Figure 4A). To achieve this, we concurrently transduced primary human T cells with two separate lentiviral vectors encoding CD33-CAR and CD70-CAR. CAR expression was once again confirmed via flow cytometry. Interestingly, expansion of CD33-CAR T cells was mildly higher than CD70-directed or co-transduced CAR T cells, though this increase is insignificant (Figure 4B). We attribute this difference to the fact that CD70 is expressed on a minor population of T cells, which could lead to a small degree of fratricide during the expansion process (Figure S7). The cytotoxic capabilities of pooled vs co-transduced CAR T-cell strategies against our AML sublines were once again assessed by culturing each CAR T-cell product with the target cells for 24 h in varying E:T ratios. Targets were distinguished from effectors via VPD450 intracellular dye and stained with early and late apoptosis markers as described above. Flow cytometric analysis revealed no statistically significant difference in the cytotoxicity of pooled vs co-transduced CAR T cells against all of our AML sublines (Figure 4C). Pooled CAR and co-transduced CAR T cells were equally able to inhibit the growth of CD33+/CD70−, CD33−/CD70+, and CD33+/CD70+ blasts in a 24 h co-culture assay with heterogeneous Molm-13 cells (Figure 4D and Figure S8). A primary AML patient sample was subsequently co-cultured in a 1:1 ratio for only 4 h with each CAR T-cell product, revealing superior cytotoxicity of our co-transduced CAR T cells compared to all other groups (Figure 4E).

### 3.5. Immunophenotypic and Functional Characterization of Co-Transduced CD33/CD70 CAR T Cells

**Figure 5:**
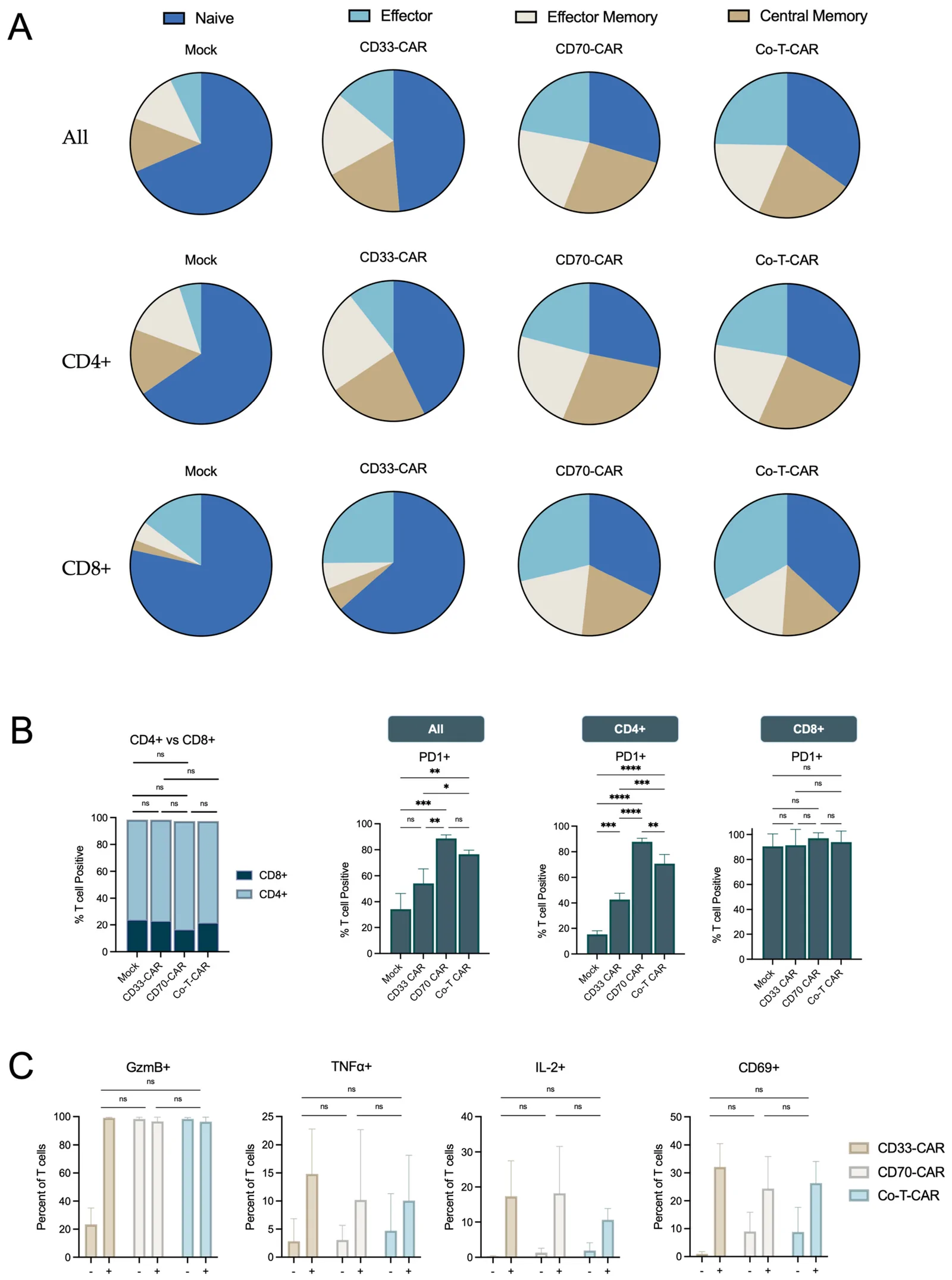
Immunophenotypic and functional characterization of single- vs co-transduced CAR T cells. (**A**) Pie charts depicting naive, effector, effector memory, and central memory landscape of individually transduced CAR T cells compared to co-transduced CAR T cells. Analysis was divided by CD4 and CD8 T-cell subsets for each CAR T-cell group. (**B**) CD4 and CD8 subsets shown for each group; PD-1 expression for all CAR T cells and CD4/CD8 subsets was measured via flow cytometry. *n* = 3 experimental replicates, three biological donors. Statistical analysis represents a one-way ANOVA with a post hoc Tukey’s test (**** *p* < 0.0001; *** *p* < 0.001; ** *p* < 0.01; * *p* < 0.05, ns—non-significant). (**C**) Flow cytometric analysis of surface CD69, as well as intracellular TNFα, IL-2, and GzmB of individually transduced CAR T cells compared to co-transduced CAR T cells with and without 24 h exposure to Molm-13 target cells. Statistical analysis represents a one-way ANOVA with a post hoc Tukey’s test (ns—non-significant).

We next sought to evaluate whether transduction with two different lentiviral vectors and subsequent expression of two different CARs had an impact on the fitness of our co-transduced CAR T cells. The differential expression of the surface markers CD45RA and CCR7 are well-established means of characterizing human T cells as naive, central memory, effector memory, and/or effector cells [50]. We found no significant phenotypic differences in individually transduced CD70-CAR T cells compared to co-transduced CAR T cells; however, there was a significantly higher percentage of naive cells in individually transduced CD33-CAR T cells (Figure 5A). Similarly, all our single- and dual-targeting CAR T-cell products had a CD4:CD8 ratio comparable to mock, untransduced primary T cells (Figure 5B). Interestingly, CD70-CAR T cells and co-transduced CAR T cells exhibited increased PD-1 expression when compared to individually transduced CD33-CAR T cells, signifying strong T-cell activation during the expansion stage of these CAR T-cell products without the presence of antigen targets (Figure 5B) [51]. Through subset analysis, the increased PD-1 expression was mainly attributed to the CD4 population, as PD-1 expression was consistent in the CD8 subset for all four CAR T-cell groups. To further delineate differences in our pooled vs co-transduced CAR T-cell products, we stimulated CAR T cells with Molm-13 AML targets at a 1:1 E:T ratio for 24 h and quantified expression of the well-described T-cell activation marker CD69, the cytolytic mediator granzyme B (GzmB), and the pro-inflammatory cytokine tumor necrosis factor alpha (TNFα) via flow cytometry. Mock T cells exhibited negligible expression of CD69, GzmB, and TNFα, with and without the presence of target cells. CD33-CAR T cells exhibited the highest increase in CD69, GzmB, and TNFα upon addition of target cells, while the CD70-CAR T cells and co-T-CAR T cells showed high baseline expression of CD69, GzmB, and TNFα regardless of the addition of target cells, which again signifies constitutively potent and activated CAR T cells when considering their high baseline level of PD-1 expression during expansion (Figure 5C and Figure S9). Altogether, these results show that the phenotype, activation, exhaustion, and cytokine release signatures of our co-transduced strategy were consistently similar to the CD70-CAR T-cell product. These results suggest that CD33/CD70 CAR co-transduction does not have a negative impact on T-cell fitness, especially when compared to similar single-antigen CAR T-cell products.

### 3.6. Co-Transduced CD33/CD70 CAR T Cells Are Superior to Pooled CAR T Cells in an Aggressive In Vivo Heterogeneous AML Model

**Figure 6:**
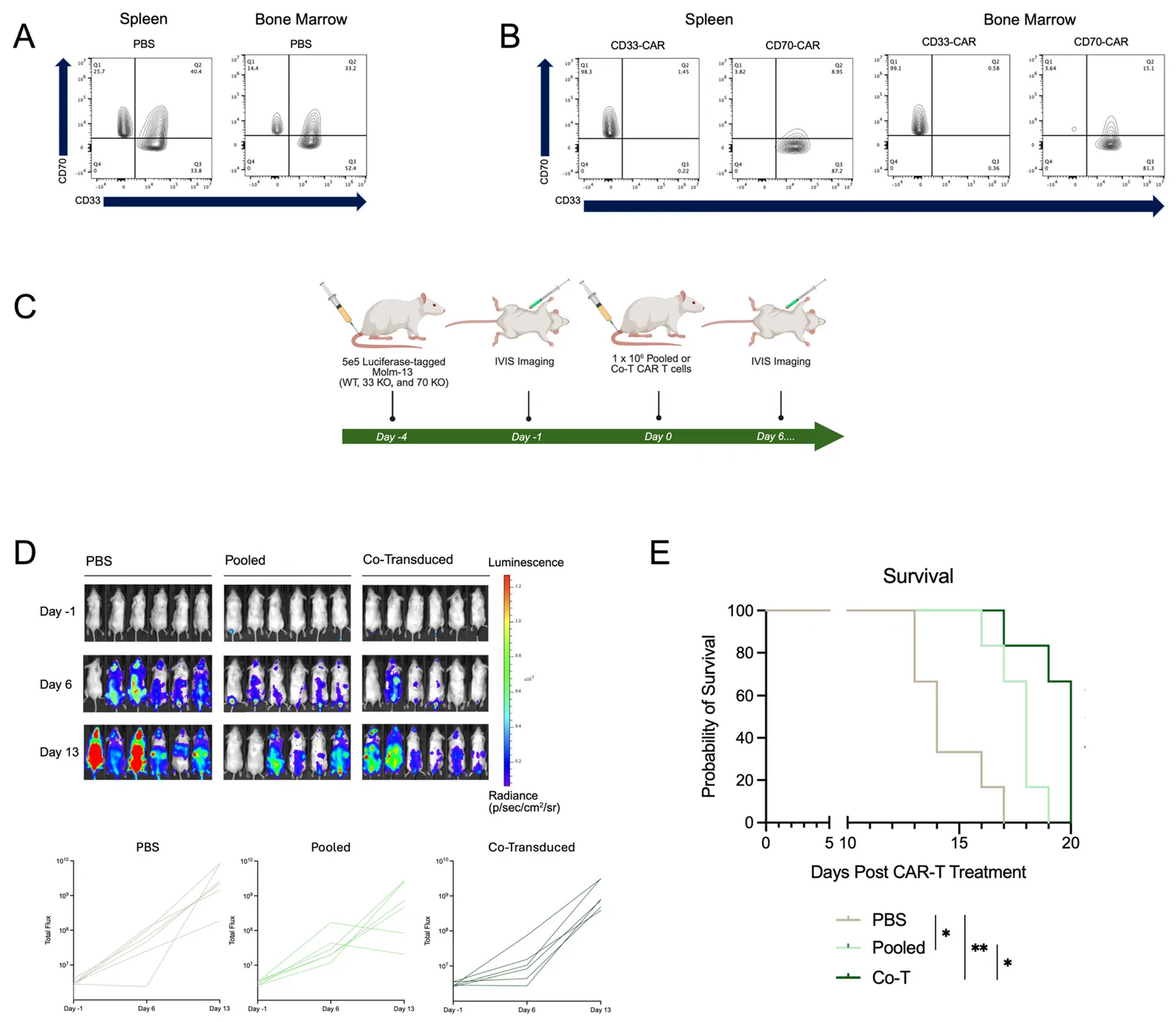
*In vivo* evaluation of CAR T-cell products in murine xenografts and flow cytometric evaluation of circulating blast subtypes following treatment. (**A**) Bone marrow and spleen endpoint analysis of heterogeneous murine AML xenograft model. NSG mice were injected with 5 × 10^5^ Molm-13 cells, of which ^1^/_3_ were CD33 KO, ^1^/_3_ were CD70 KO, and ^1^/_3_ were WT. Flow cytometry plots depicting CD33 and CD70 surface expression of human leukemia cells in spleen and bone marrow are shown. (**B**) Mice engrafted with heterogeneous Molm-13 AML cells were intravenously injected with 1 × 10^6^ CD33-CAR T cells or CD70-CAR T cells four days post tumor injection. Flow cytometry analysis of harvested spleen and bone marrow was done at endpoint to assess CD33 and CD70 expression in leukemia cells. (**C**) Experimental schema for assessment of dual CD33/CD70 CAR T-cell strategy in a heterogeneous AML model. Six NSG mice per group were intravenously injected on day −4 with 5 × 10^5^ luciferase-expressing Molm-13 cells, of which ^1^/_3_ were CD33 KO, ^1^/_3_ were CD70 KO, and ^1^/_3_ were WT. Mice were intraperitoneally injected with D-luciferin and imaged via IVIS on day −1 and weekly thereafter for monitoring tumor burden via bioluminescence. On day 0, mice were intravenously injected with either PBS, 1 × 10^6^ pooled CAR T cells, or co-transduced CAR T cells. (**D**) Bioluminescence under IVIS with corresponding total flux quantified in standard radiance total flux units (p/sec/cm^2^/sr). (**E**) Kaplan–Meier survival curves of mice treated with PBS, pooled CAR T cells, or co-transduced CAR T cells. Statistical analysis represents Log-Rank (Mantel–Cox) test with Bonferroni correction. (** *p* < 0.01; * *p* < 0.05).

To create a heterogeneous murine AML xenograft model, male and female NSG mice aged between 8 and 12 weeks were intravenously injected with 5 × 10^5^ Molm-13 cells, of which 1/3 were CD33 KO, 1/3 were CD70 KO, and 1/3 were WT. Upon endpoint, bone marrow and spleen were harvested. Flow cytometry analysis showed an even spread of CD33+/CD70−, CD33−/CD70+, and CD33+/CD70+ cell populations (Figure 6A), confirming it to be a heterogeneous *in vivo* AML model. Next, to establish that our model was appropriate to study antigen escape secondary to tumor heterogeneity, we treated the mice with our single-antigen-directed CAR T cells. Mice were first injected with the heterogeneous Molm-13 tumor injection, and four days later were intravenously injected with 1 × 10^6^ CD33-CAR T cells or 1 × 10^6^ CD70-CAR T cells. Upon endpoint, mice from each group were dissected and spleen and bone marrow were harvested. Mice treated with CD33-CAR T cells contained a majority of CD70+ AML cells in the spleen and bone marrow, while mice treated with CD70-CAR T cells alone contained a majority of CD33+ AML cells, confirming this was an excellent model to study antigen heterogeneity in the context of CAR targeting (Figure 6B). These findings confirm that single-targeting CARs are insufficient to eradicate all heterogeneous AML blast populations.

After establishing our heterogeneous AML xenograft model, we next evaluated which dual CD33/CD70 CAR T-cell strategy would provide a better survival advantage. Once again, male and female NSG mice aged between eight and 12 weeks were intravenously injected with 5 × 10^5^ luciferase-expressing Molm-13 cells, of which 1/3 were CD33 KO, 1/3 were CD70 KO, and 1/3 were WT (day −4). Three days later, mice were intraperitoneally injected with luciferin and imaged using the In Vivo Imaging System (IVIS) to confirm leukemia engraftment (day −1). The next day (day 0), mice were intravenously injected with either PBS, 1 × 10^6^ pooled CAR T cells, or 1 × 10^6^ co-transduced CAR T cells. Tumor burden was assessed weekly via bioluminescence imaging (Figure 6C). IVIS imaging showed improved cancer cell clearance and delayed flux of co-transduced CAR T cells compared to pooled CAR T cells (Figure 6D). Day 13 was the final IVIS imaging timepoint, as animals began reaching natural and/or humane endpoints shortly afterwards. Both dual-CAR T-cell strategies had a significant survival advantage over the PBS group, with the co-transduced CAR T-cell group having an additional survival advantage over the pooled CAR T-cell group in both median survival and Kaplan–Meier survival analyses (Figure 6E).

## 4. Discussion

In this study, we investigated the feasibility and efficacy of a dual-targeting CD33/CD70-CAR T-cell approach against a heterogeneous AML model both *in vitro* and *in vivo*. To this end, we designed and evaluated multiple CAR T-cell products, including single-antigen CAR T cells against CD33 or CD70; pooled CAR T cells comprising a combination of single-target CD33 and CD70 CAR T cells in a 1:1 ratio; and a co-transduced CAR T-cell product, consisting of a single population of T cells expressing CD33-CARs and CD70-CARs. CRISPR-Cas9 was used to knock out either CD33 or CD70 in Molm-13 AML cells in order to recapitulate tumor heterogeneity. We have demonstrated the potent cytotoxic ability of multiple CAR T-cell products against well-characterized AML cell lines and patient samples, and that a dual-targeting CAR T-cell strategy can prolong survival in an aggressive and heterogeneous murine AML xenograft model.

CD33, originally known as the MY9 antigen, was discovered as a surface marker for myeloid-derived leukemic stem cells and blasts, as well as healthy myeloid progenitors and monocytes, in 1983 [52]. Years later, MY9 became formally known as sialic acid binding Ig-like lectin 3 (siglec-3; CD33) and was characterized functionally as an inhibitory immune regulator that functions through its immunoreceptor tyrosine-based inhibitory motif (ITIM) [53]. In 2000, the FDA approved gemtuzumab ozogamicin (Mylotarg; GO), an anti-CD33 monoclonal antibody conjugated to a cytotoxic calicheamicin derivative [54]. Since its approval, GO has become one of the few directed standard-of-care therapies in the treatment of *de novo* and R/R AML [55]. Additionally, CD33 has been extensively evaluated as an AML CAR target, both preclinically and clinically, given its surface expression in ~85% of AML cases [56,57,58]. Several clinical trials in the United States (ClinicalTrials.gov identifier: NCT03126864, NCT03927261, NCT03971799, NCT04835519, NCT05672147, NCT05984199, NCT05945849) have demonstrated limited success of CD33-CAR T-cell therapy in AML. As a whole, this collection of phase I and phase II clinical trials reveals that patients can tolerate CD33-CAR T-cell therapy at clinically relevant cell doses. While a minority of patients achieved remission following treatment with CD33-CAR T-cell products, this was inconsistent and unpredictable. Multiple groups have evaluated the challenges associated with CAR T-cell therapy for AML, and antigen escape post-treatment has been identified as a major limitation [21,33,35,59]. This is unsurprising given the well-established phenomenon of CD19-negative relapse in B-ALL patients treated with CD19-directed CAR T-cell therapies [60,61]. While CD33 is not expressed on pluripotent hematopoietic stem cells, it is expressed on other hematopoietic precursor cells. This has led to on-target off-tumor toxicity in prior CD33-CAR clinical trials and is a known limitation as evidenced by monocytopenia, neutropenia, and thrombocytopenia noted in patients treated with CD33-directed CAR T-cell products. However, despite the known myelotoxic effects of CD33-based CARs, several patients have achieved complete remission with hematologic recovery [56,57,62,63]. Additionally, patients with R/R AML are destined for HSCT, which ameliorates the concern for transient cytopenias in this patient population.

CD70 was first discovered in 1993 as a ligand for the T- and B-cell marker CD27, and, in later years, was characterized functionally as an immune activator expressed on healthy lymphocytes. In AML, however, CD70 is known to be variably overexpressed on AML cell lines and not expressed in hematopoietic stem and precursor cells [29,64,65,66]. Preclinically, CD70 has been identified as an attractive AML target given its expression on AML blasts and lack of surface expression on hematopoietic stem cells (HSCs) [30,31,34,38,49,67]. This lack of expression on healthy myeloid progenitor cells limits myeloid toxicity resulting from on-target off-tumor CAR T-cell activity [30,36]. More recently, CD70 has been established as a viable CAR target clinically for AML (ClinicalTrials.gov identifier: NCT06326463), as well as multiple other tumor types, including T- and B-cell malignancies, renal cell carcinoma, and glioblastoma. Overall, the therapeutic results for single-targeting CAR T-cell therapies for AML are highly variable due to tumor heterogeneity and selective antigen pressure, which leads to the growth of antigen-negative blast populations [28,68]. Antigen-negative relapse is well-characterized in B-ALL following CD19-directed CAR T-cell therapy, and the concern for antigen-negative relapse is even greater in myeloid malignancies, such as AML, given the inherently heterogeneous tumor landscape [35,60,69,70,71].

In 2020, Perna et al. utilized transcriptomics and proteomics datasets from AML samples in an attempt to identify AML CAR antigens that could be targeted in combination with already established AML CAR antigens [36]. They identified multiple combinations of AML surface antigens in which their surface expression exceeded that of either antigen by itself on primary AML samples. Since then, dual-targeting CAR T cells have emerged as a potential strategy to overcome antigen heterogeneity and escape in AML, and dual-targeting CAR T cells have exhibited superiority to single-targeting CAR T cells in preclinical models [72,73,74]. Preclinical dual-CAR targets for AML include CD33/CLL1, CD123/CLL1, CD33/CD123, and ADGRE2/CLEC12A, with ADGRE2/CLEC12A now being evaluated clinically in the US (NCT05748197) [33,72,75,76,77]. Interestingly, one dual-antigen combination established by Perna et al.—CD33/CD70—has yet to be explored as a dual-CAR product [36]. Recently, Silva et al. validated CD33 and CD70 as dual targets through the development of a CD70-CAR T cell that secretes a CD33/CD3-directed bispecific T-cell-engaging antibody molecule (TEAM; 70^33^) [38]. This group demonstrated that 70^33^ T cells exhibit superior cytotoxicity both *in vitro* and *in vivo* compared to a CD70-CAR T cell that secretes a CD19/CD3-directed TEAM (70^19^) against a heterogeneous AML model. This preclinical study confirms that the combination targeting of CD33 and CD70 is a viable strategy to effectively address a heterogeneous malignancy, such as AML. There is no evidence that any on-target off-tumor toxicity exhibited by CD33-CARs will be exacerbated by the additional targeting of CD70 due to the lack of CD70 expression on HSCs. Sauer et al. specifically demonstrated that CD70-CAR T cells did not cause HSC death in direct co-culture assays [30]. In fact, multiple studies depicting the expression profile of both CD33 and CD70 have shown a distinct lack of healthy tissue overlap [30,36,78]. Therefore, by selecting CD70 as our second target, we limit further safety concerns, and any potential concerns about myeloablation can be ameliorated by impending transplant.

With the advent of directed therapies, selective antigen pressure leading to the survival of antigen-negative clones is a concern, particularly in patients with R/R AML who have been exposed to multiple therapies. In particular, the widely used CD33-directed immunotherapy GO could lead to negative selection of CD33+ AML blasts [5,55]. We subsequently sought to confirm that CD33 and CD70 gene expression did not decrease in relapsed AML samples. After categorizing patient samples by diagnosis vs relapse and assessing gene expression via bulk RNA sequencing datasets, no differences in CD33’s or CD70’s gene expression were noted. A limitation of bulk RNA sequencing is that it is not entirely representative of surface protein expression, and additionally, it cannot differentiate antigen heterogeneity from blast to blast within a single population. To characterize surface protein expression of CD33 and CD70, we analyzed primary AML samples by flow cytometry and found heterogeneous expression of CD33 and CD70 surface markers on all samples. We recapitulated this heterogeneity using CRISPR-Cas9 in the well-characterized AML Molm-13 cell line. While expression of CD33 and CD70 can be plastic throughout the course of the disease, this knockout model more closely mimics clinical relevance than wildtype cell line models and is supported by the past literature examining CD33/CD70-directed therapeutic approaches [38].

We designed pooled and co-transduced CAR T cells using CD33 and CD70-directed scFvs. We chose to use the M195 clone anti-CD33 scFv due to its superior efficacy when compared directly to other CD33-CAR scFv clones [58]. In CD70-CARs, the majority of designed products consist of a natural receptor (CD27) binding domain, whereas our study utilizes a CD70-scFv-based CAR. The use of an scFv-based design allows for development of future dual-CAR iterations with a tandem scFv design [79]. CD28 and 4-1BB are the two most commonly utilized co-stimulatory domains seen in CAR T-cell designs, with CD28 being associated with increased cytotoxic capabilities and 4-1BB with increased CAR T-cell persistence [80]. We chose to utilize CD28 in our constructs given the aggressiveness of AML and the fact that patients will likely proceed to transplant soon after CAR T-cell therapy. This negates the desire for CAR T cells with a persistent phenotype and thus makes CD28 the first choice for our constructs. Our pooled CAR T-cell product comprised CAR T cells individually transduced with CD33-CAR *or* CD70-CAR lentiviral vectors and subsequently combined in a 1:1 ratio, while our co-transduced CAR T-cell product was developed by transducing T cells simultaneously with CD33-CAR *and* CD70-CAR lentiviral vectors. With a co-transduced strategy, it is possible to have a heterogeneous population of CAR T cells, in which subsets of transduced T cells may express only one of the desired CAR constructs or none of the desired CAR constructs. This is consistent with other CAR T-cell products, which contain a range of CAR expression levels [81]. There is also evidence that CAR T cells, activated through their CARs, clonally expand exponentially *in vivo*, a phenomenon that could naturally select for the most useful CAR T cell [82]. However, we do acknowledge that the presently designed CAR constructs do not allow for identification of distinct populations of single-CAR-positive versus dual-CAR-positive cells within the co-transduced group. While CD33-CAR expression can be assessed using a CD33 Fc protein conjugated to a fluorophore, CD70-CAR does not have that luxury, as T cells naturally express its binding partner, CD27 [65,83]. Prior CD70-CAR groups have incorporated reporter genes into their construct to overcome this limitation; however, that is not a direct measure of CAR expression [38]. To overcome this, our future dual-targeting CAR studies will include distinct linker regions for each scFv.

There is concern that baseline CD70 expression on primary human T cells could lead to fratricide in the manufacturing of CD70-CAR T cells [84,85]. Although we demonstrated low levels of CD70 expression on primary human T cells, this did not lead to significant fratricide during the expansion process. Additionally, prior groups have shown CD70-CAR success without CD70 knockout [30,31,38]. For these reasons, we chose to proceed with our current study without generating CD70-negative T cells. We did, however, note increased baseline levels of PD-1 with T cells expressing CD70-CAR, likely due to exposure to their cognate antigen during the manufacturing process. To elucidate the role of CD70 baseline expression on dual-targeting CD70-CAR T cells, future studies will knockout CD70 using CRISPR-Cas9 in healthy T cells prior to CAR generation.

Interestingly, transducing primary human T cells in our co-T group with double the amount of total lentiviral vector did not negatively impact their activation nor their phenotype when compared to singly transduced T cells, and, ultimately, AML murine xenografts treated with co-T-CAR T cells exhibited superior survival over those treated with pooled CAR T cells. We suspect that this finding can be attributed to the increased *in vivo* expansion of our co-T-CAR T cells when activated by either CD33 or CD70. While the present study highlights the merits of a co-transduced approach, there are limitations. A co-transduced product requires two CAR viral vectors, which can lead to higher production costs. Additionally, a two-vector approach can lead to product heterogeneity and unpredictable vector copy number, which could raise concerns in terms of clinical translation. Bicistronic and tandem dual-targeting CAR approaches could ameliorate some of the above concerns but may have disadvantages of their own such as decreased CAR expression [86]. We will continue to evaluate the optimal dual-targeting approach in future studies by comparing co-transduced dual-targeting CD33/CD70 CAR T cells with bicistronic and tandem approaches. We will also study differences in using either CD28 or 4-1BB co-stimulatory domains, or a combination of both in our bicistronic designs as we will look to further optimize our dual-CAR approach. Lastly, future studies will utilize multiple different AML cell lines, as well as more primary patient samples *in vitro*, to assess the functionality of our CARs. Our current cell line murine model was extremely aggressive and did not allow for durable remission in any group. Utilizing a patient-derived xenograft murine model will allow for greater differences in survival and CAR T-cell persistence to become apparent.

## 5. Conclusions

In summary, we describe two dual-targeting CAR T-cell products for AML capable of targeting CD33 and CD70 simultaneously. We have demonstrated superior cytotoxic ability of the pooled CAR and co-transduced CAR products compared to single-targeting CAR T cells *in vitro* and have additionally shown that a co-transduced dual-targeting approach is more effective compared to a pooled CAR approach *in vivo*. The present study’s evaluation of a dual CD33/CD70-CAR T-cell product will lay the foundation for further preclinical analyses to determine the optimal CD33/CD70-CAR approach for eventual clinical translation.

## Data Availability

Data used to draw conclusions in this manuscript are available in the article and the Supplementary Materials. Raw data are available upon request to the corresponding author.

## Supplementary Materials

The following supporting information can be downloaded at: https://www.mdpi.com/article/10.3390/cancers18193183/s1, Figure S1: Gating strategy of CD33/CD70 surface expression via flow cytometry on primary AML cells; Figure S2: Gating strategy of CD33/CD70 surface expression via flow cytometry on AML cell lines; Figure S3: CD33/CD70 Molm-13 fluorescence-activated cell sorting (FACS) strategy; Figure S4: Gating strategy of CAR surface expression via flow cytometry; Figure S5: Gating strategy for target cells in flow cytometry-based cytotoxicity assay; Figure S6: Donor variability in CD33/CD70-CAR cytotoxicity; Figure S7: Gating strategy and surface expression of CD33/CD70 via flow cytometry on donor derived primary T cells; Figure S8: Donor variability in dual CD33/CD70-CAR T-cell cytotoxicity; Figure S9: Gating strategy for intracellular flow cytometry assays assessing for activation markers and cytolytic mediators; Table S1: Cytogenetic and molecular features of primary AML patient samples utilized.
