# Supplementary material for: Developing a Dual-Targeting CD33/CD70 CAR T-Cell Strategy Against Acute Myeloid Leukemia to Overcome Tumor Heterogeneity
Source: Cancers (Basel). 2026 Oct 2;18(19):3183. doi: 10.3390/cancers18193183 (PMC13664042; doi:10.3390/cancers18193183)
Supplement: Supplementary file 1 [file cancers-18-03183-s001.zip › cancers-4589385-supplementary.pdf]

Developing a dual-targeting CD33/CD70 CAR T-cell strategy against acute myeloid leukemia to overcome tumor heterogeneity

Supplementary Material

Supplementary Figures

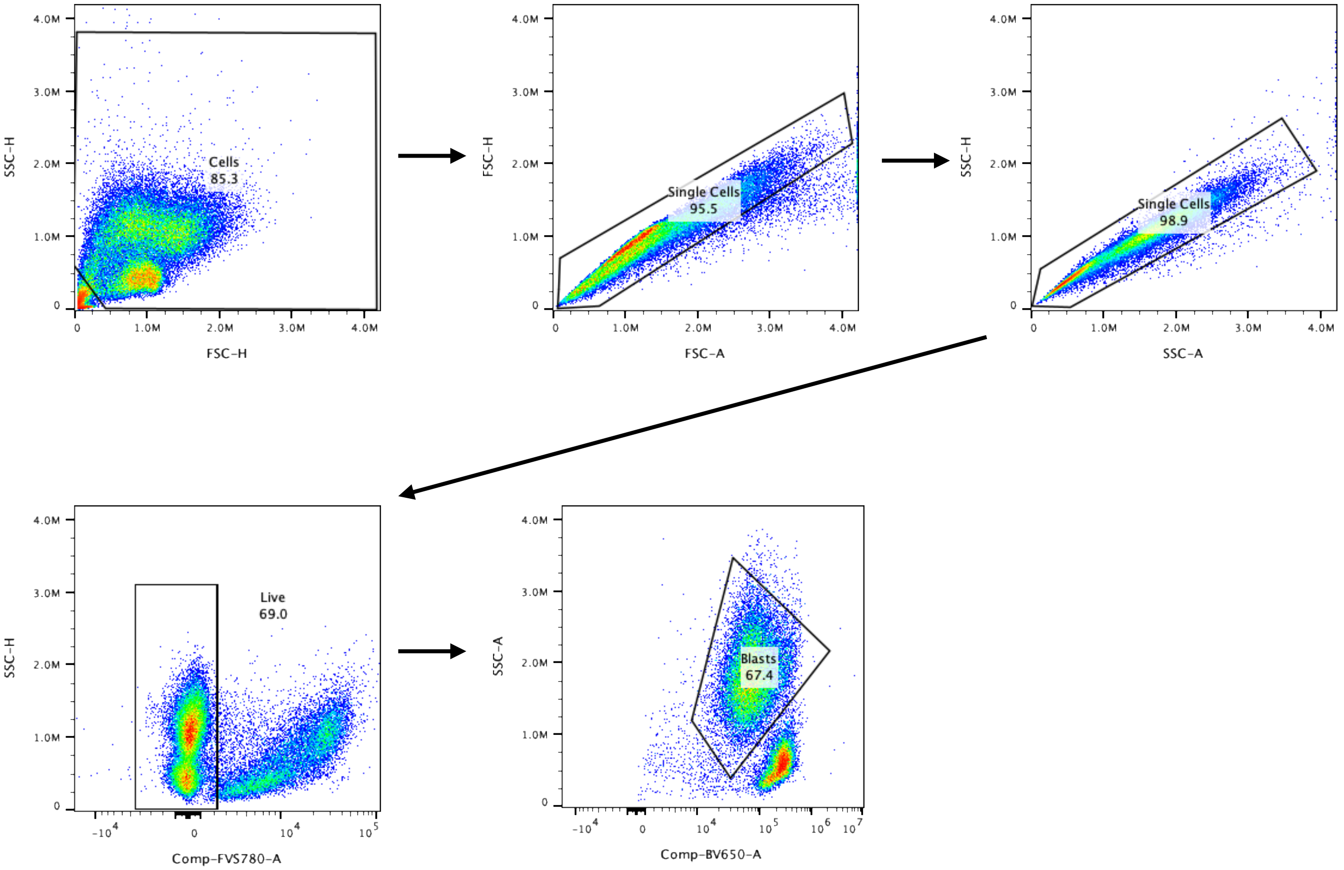

Figure S1: Gating strategy of CD33/CD70 surface expression via flow cytometry on primary AML cells. Cell debris were gated out via low side scatter, forward scatter. Doublets were then removed from the resulting subpopulation via gates as shown. eFluor 780 positive cells were gated out to obtain a subpopulation of live cells. An antibody against human CD45-BV650 in combination with high side scatter was used to isolate blasts.

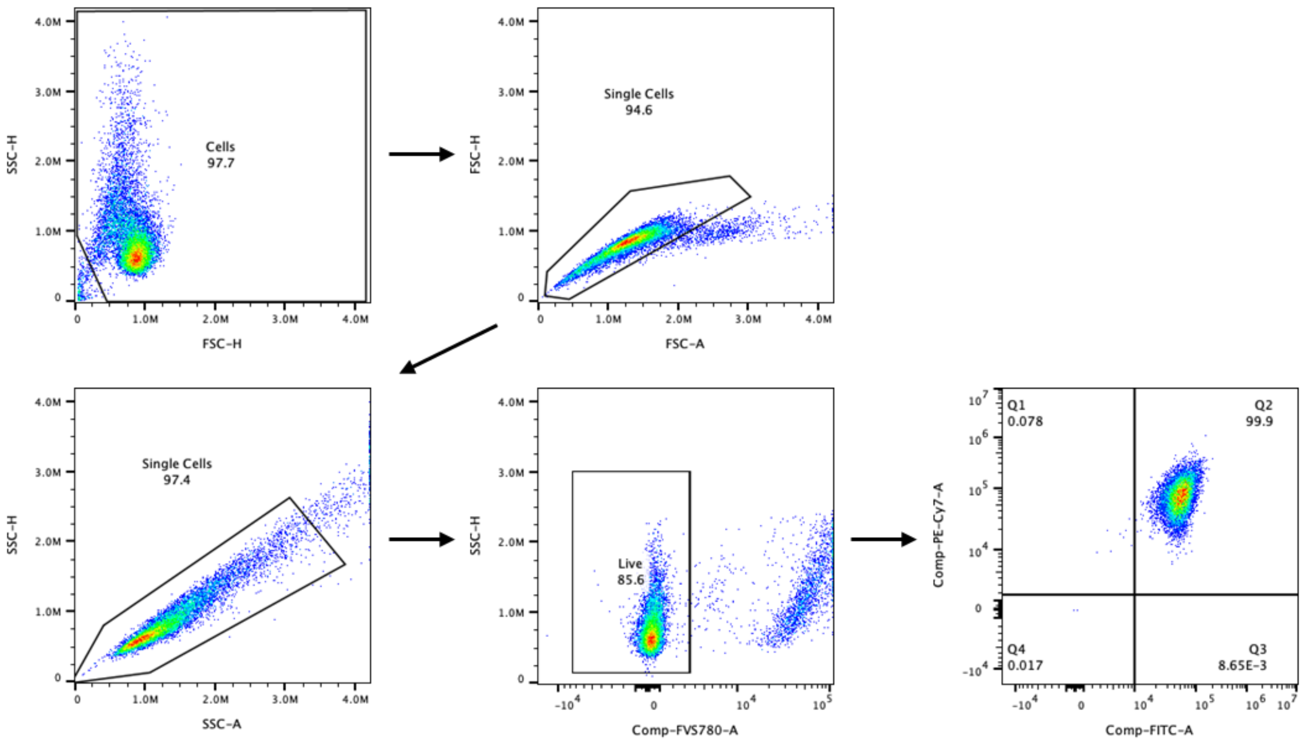

Figure S2: Gating strategy of CD33/CD70 surface expression via flow cytometry on AML cell lines. Cell debris were gated out via low side scatter, forward scatter. Doublets were then removed from the resulting subpopulation via gates as shown. eFluor 780 positive cells were gated out to obtain a subpopulation of live cells. Finally, an antibody against CD33 conjugated to FITC and an antibody against CD70 conjugated to PE-Cy7 were quantified through FITC and Pe-Cy7 quadrants as shown.

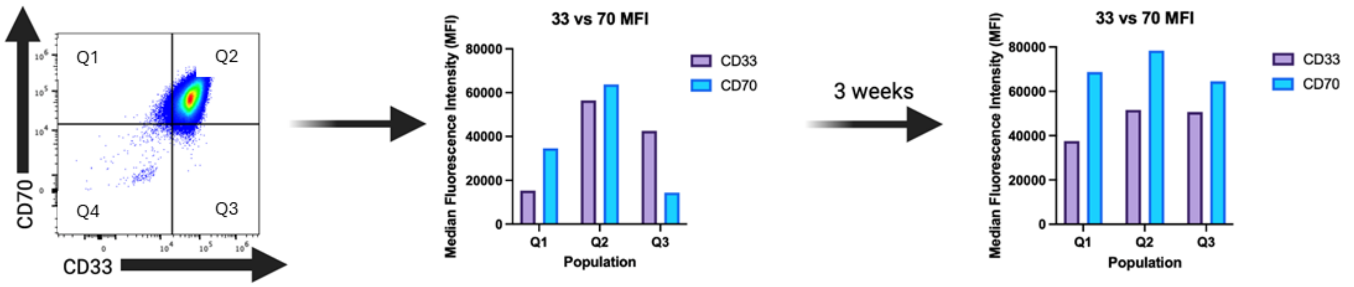

Figure S3: CD33/CD70 Molm-13 fluorescence-activated cell sorting (FACS) strategy. Molm-13 cell lines were assayed for CD33 and CD70 expression using flow cytometry with an antibody against CD33 conjugated to FITC and an antibody against CD70 conjugated to PE-Cy7. Fluorescence-Activated Cell Sorting (FACS) was used to isolate CD33 and CD70 low expressing cells into separate populations. Surface expression of CD33 and CD70 were again assayed as above 3 weeks after FACS, confirming plastic expression of both CD33 and CD70 despite initial FACS.

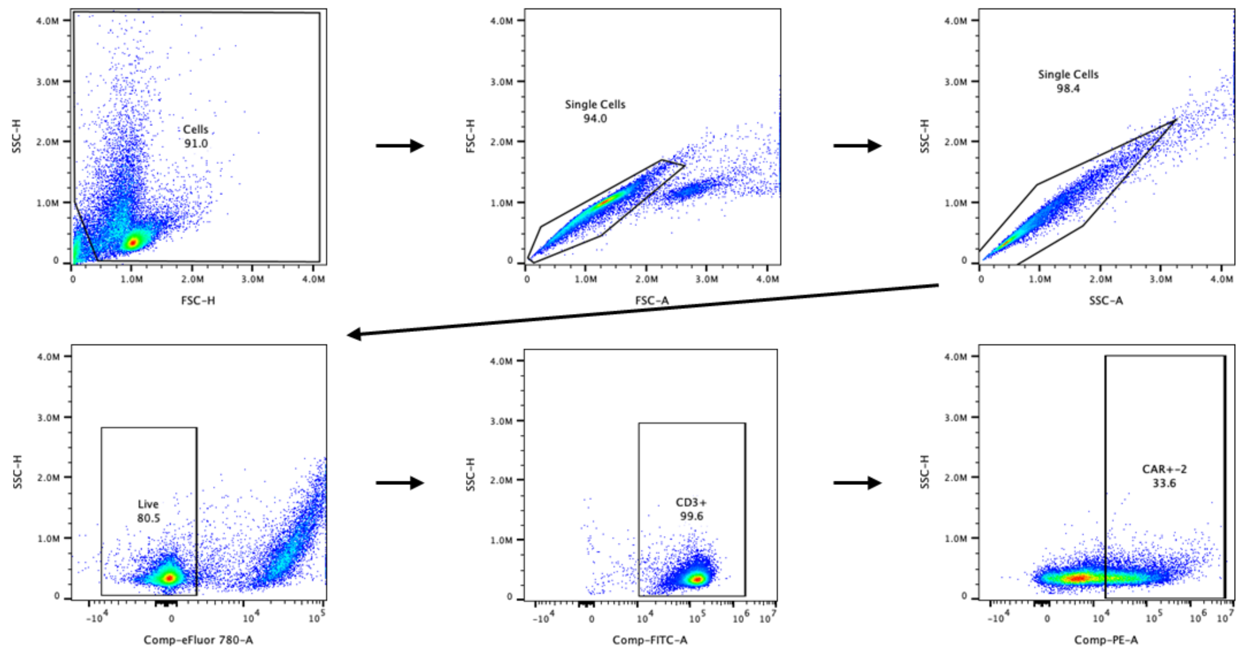

Figure S4: Gating strategy of CAR surface expression via flow cytometry. Cell debris were gated out via low side scatter, forward scatter. Doublets were then removed from the resulting subpopulation via gates as shown. eFluor 780 positive cells were gated out to obtain a subpopulation of live cells. An antibody against CD3-FITC was used to gate in T cells, and an antibody against the G4S linker connecting the variable heavy and light chains of our car constructs conjugated to the fluorophore PE was used to gate in cells expressing CAR construct.

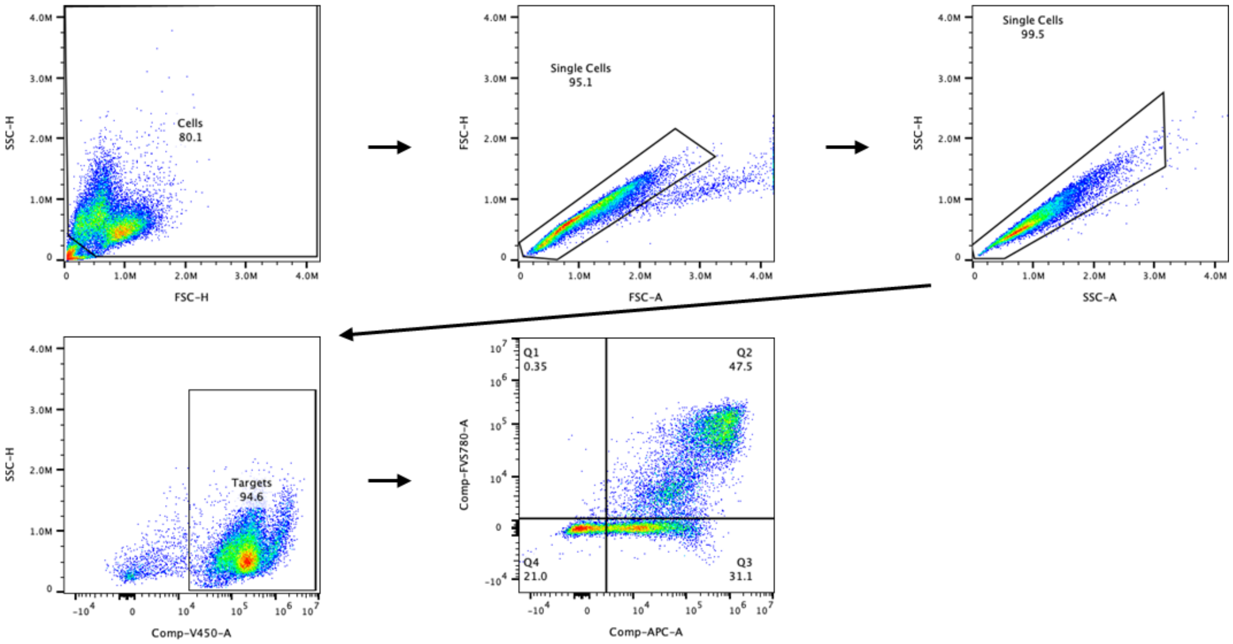

Figure S5: Gating strategy for target cells in flow cytometry-based cytotoxicity assay. Cell debris were gated out via low side scatter, forward scatter. Doublets were then removed from the resulting subpopulation via gates as shown. Target cells, previously stained with VPD450, were gated in. Quadrants measuring the dead cell marker eFluor 780 as well as the early apoptotic marker annexin-V were used to quantify cytotoxicity of target cells using quadrants as shown.

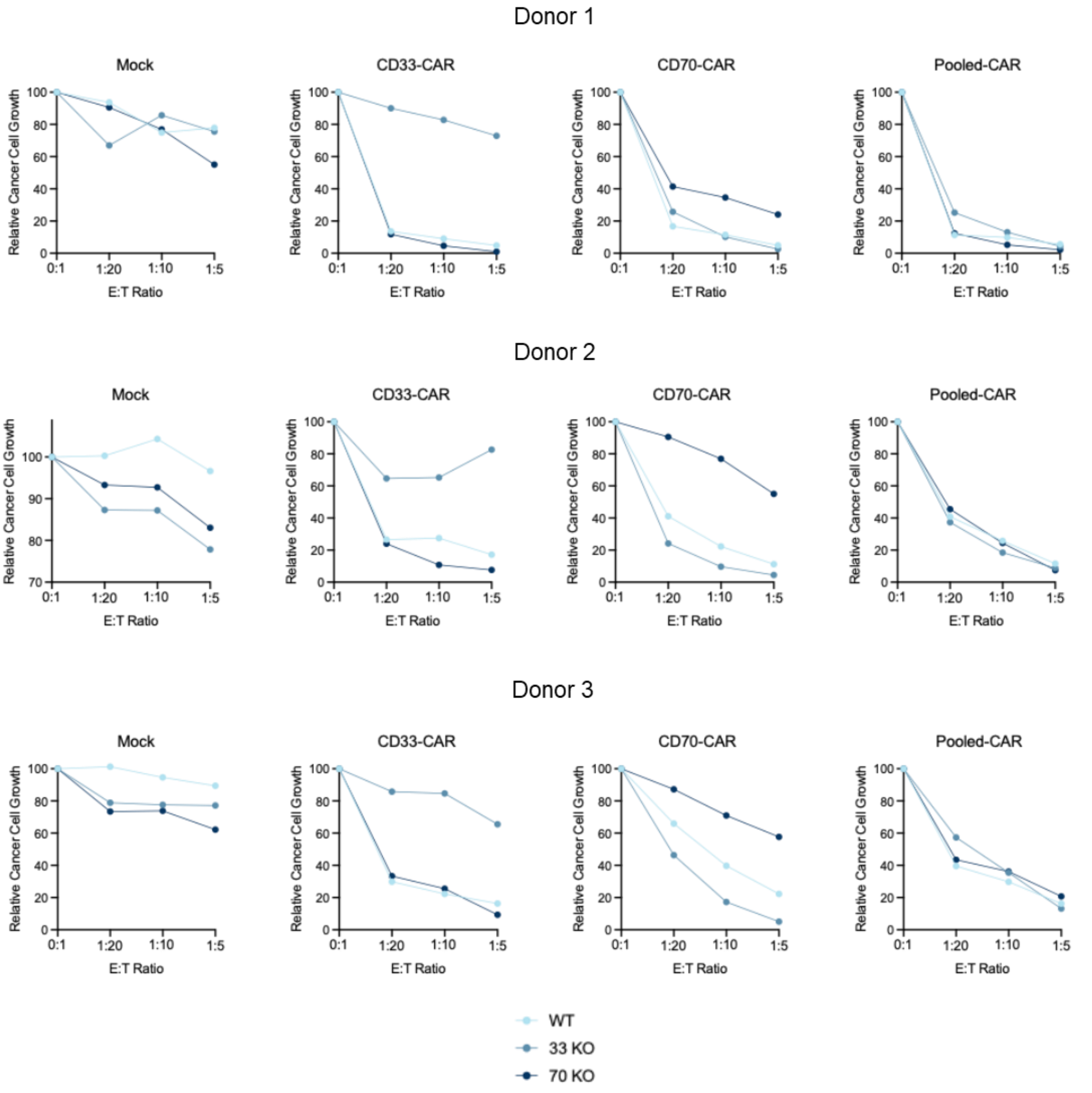

Figure S6: Donor variability in CD33/CD70-CAR cytotoxicity. Relative cell growth of WT, CD33 KO, or CD 70 KO Molm-13 AML target cells at a range of E:T ratios when co-cultured with mock, CD33-CAR, CD70-CAR and pooled CAR T cells across three different biological donors.

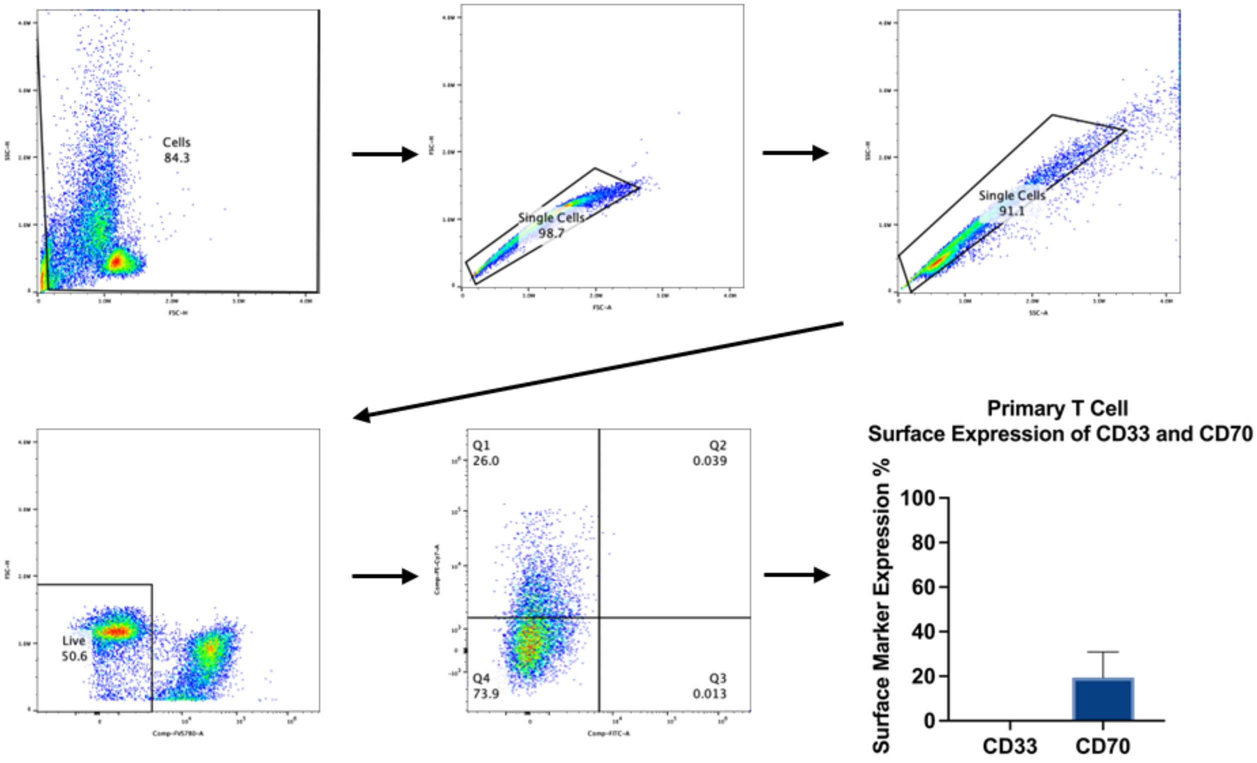

Figure S7: Gating strategy and surface expression of CD33/CD70 via flow cytometry on donor derived primary T cells. Cell debris were gated out via low side scatter, forward scatter. Doublets were then removed from the resulting subpopulation via gates as shown. Following this, eFluor 780 positive cells were gated out to obtain a subpopulation of live cells. Finally, an antibody against CD33-FITC and an antibody against CD70-PE-Cy7 were used to determine CD33 and CD70 surface expression as shown. n=3 biological donors.

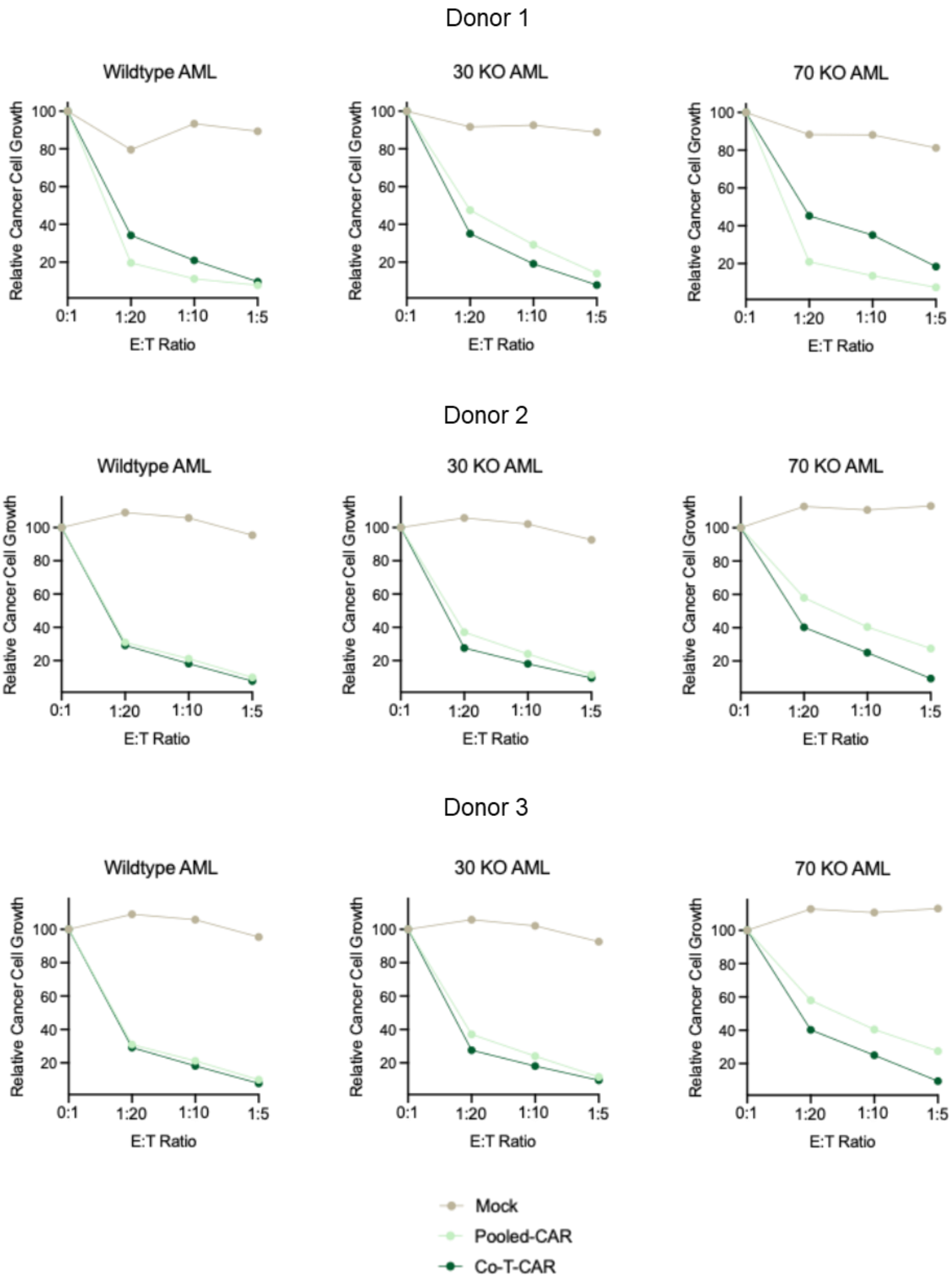

Figure S8: Donor variability in dual CD33/CD70-CAR T-cell cytotoxicity. Relative cell growth of WT, CD33 KO, or CD 70 KO Molm-13 AML target cells at a range of E:T ratios when co-cultured with mock, pooled and Co-T-CAR T cells across three different biological donors.

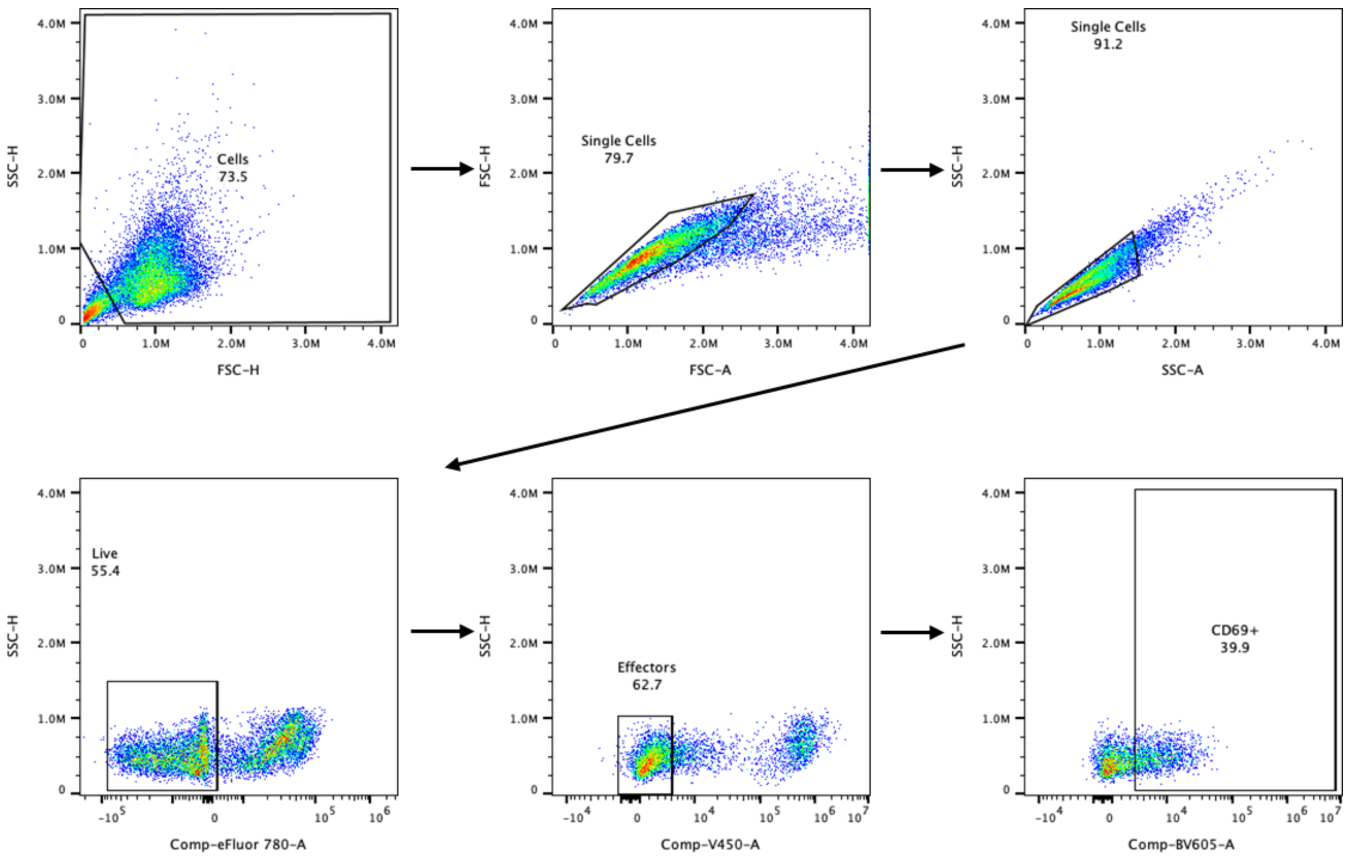

Figure S9: Gating strategy for intracellular flow cytometry assays assessing for activation markers and cytolytic mediators. Cell debris were gated out via low side scatter, forward scatter. Doublets were then removed from the resulting subpopulation via gates as shown. eFluor 780 positive cells were gated out to obtain a subpopulation of live cells. Target cells, previously stained with VPD450, were gated out to isolate a population of T cells.

Supplementary Table

Table S1: Cytogenetic and molecular features of primary AML patient samples utilized

Donor	-5/del(5q)	-7/del(7q)	t(8;21)	t(16;16)(p13.1q22)	KMT2A-R	FLT3-ITD	CEBPA mutation	FLT3 D835 mutation	NPM mutation	C-kit mutation	JAK2 mutation	AML WHO Classification
1	Negative	Negative	Negative	Positive	Negative	Negative	Negative	Negative	Negative	Positive	Negative	Acute myeloid leukemia with inv(16)(p13.1q22) or t(16;16)(p13.1;q22)
2	Negative	Negative	Negative	Negative	Negative	Negative	Negative	Unknown or N/A	Negative	Unknown or N/A	Unknown or N/A	Acute myeloid leukemia with myelodysplasia-related changes
3	Negative	Negative	Negative	Negative	Positive	Negative	Negative	Unknown or N/A	Negative	Unknown or N/A	Unknown or N/A	Acute myeloid leukemia with KMT2A-R
